# Characterization of the direct targets of FOXO transcription factors throughout evolution

**DOI:** 10.1111/acel.12479

**Published:** 2016-04-08

**Authors:** Ashley E. Webb, Anshul Kundaje, Anne Brunet

**Affiliations:** ^1^ Department of GeneticsStanford University300 Pasteur DriveStanfordCA94305USA; ^2^ Glenn Laboratories for the Biology of Aging at StanfordStanfordCA94305USA; ^3^ Present address: Department of Molecular Biology, Cell Biology and BiochemistryBrown UniversityProvidenceRI02903USA

**Keywords:** FOXO, DAF‐16, ChIP‐seq, evolutionary conservation, transcriptional networks

## Abstract

FOXO transcription factors (FOXOs) are central regulators of lifespan across species, yet they also have cell‐specific functions, including adult stem cell homeostasis and immune function. Direct targets of FOXOs have been identified genome‐wide in several species and cell types. However, whether FOXO targets are specific to cell types and species or conserved across cell types and throughout evolution remains uncharacterized. Here, we perform a meta‐analysis of direct FOXO targets across tissues and organisms, using data from mammals as well as *Caenorhabditis elegans* and *Drosophila*. We show that FOXOs bind cell type‐specific targets, which have functions related to that particular cell. Interestingly, FOXOs also share targets across different tissues in mammals, and the function and even the identity of these shared mammalian targets are conserved in invertebrates. Evolutionarily conserved targets show enrichment for growth factor signaling, metabolism, stress resistance, and proteostasis, suggesting an ancestral, conserved role in the regulation of these processes. We also identify candidate cofactors at conserved FOXO targets that change in expression with age, including CREB and ETS family factors. This meta‐analysis provides insight into the evolution of the FOXO network and highlights downstream genes and cofactors that may be particularly important for FOXO's conserved function in adult homeostasis and longevity.

## Introduction

FOXO transcription factors (FOXOs) are evolutionarily conserved regulators of longevity and are inhibited by the insulin/insulin‐like growth factor (IGF) signaling pathway (Kenyon, 2010). In worms, deficiency in the insulin receptor (*daf‐2*), which leads to FOXO/DAF‐16 activation, more than doubles worm lifespan, and this extension is FOXO/DAF‐16 dependent (Kenyon *et al*., 1993; Lin *et al*., 1997; Ogg *et al*., 1997). Similarly, mutations in the *Drosophila* insulin‐like receptor (*InR*) or the insulin receptor substrate protein (*chico*) activate the *Drosophila* FOXO ortholog (dFOXO) and extend lifespan by 85% and 48%, respectively (Clancy *et al*., 2001; Tatar *et al*., 2001). In mice, conditions that activate FOXO factors, such as reduced IGF1 or insulin signaling, also increase lifespan by 16–33% (Bluher *et al*., 2003; Holzenberger *et al*., 2003). The contribution of the four mammalian FOXO family members (FOXO1, 3, 4, and 6) to longevity in mammals has not been fully investigated, but *Foxo3* is required for lifespan extension in response to dietary restriction in the mouse (Shimokawa *et al*., 2015). Recent evidence has linked FOXO to lifespan in humans as well, as single‐nucleotide polymorphisms (SNPs) in the *FOXO3* locus have been associated with exceptional longevity in several independent cohorts (Morris *et al*., 2015). Together, these findings implicate FOXOs as evolutionarily conserved central regulators of longevity.

In mammals, FOXO factors are expressed broadly, including in the brain, immune system, liver and muscle, and are implicated in several tissue‐specific processes (Biggs *et al*., 2001; Salih & Brunet, 2008). For example, FOXOs regulate tissue‐specific stem cell homeostasis, including neural, muscle, and hematopoietic stem cell maintenance in the adult (Miyamoto *et al*., 2007; Tothova *et al*., 2007; Paik *et al*., 2009; Renault *et al*., 2009; Gopinath *et al*., 2014). In addition to regulating stem cell compartments, FOXOs modulate immune function, such as T‐cell tolerance, glucose and lipid metabolism in the liver, and cellular quality control in muscle, cardiomyocytes, and neurons (Matsumoto *et al*., 2007; Ochiai *et al*., 2012; Ouyang *et al*., 2012; Kim *et al*., 2013; Haeusler *et al*., 2014; Webb & Brunet, 2014). While significant progress has been made toward understanding FOXOs function in different mammalian tissues, precisely how FOXOs perform cell type‐specific functions remains unknown.

Direct targets of FOXO have been identified genome‐wide in worms, flies, mouse, and human cells by chromatin immunoprecipitation followed by direct sequencing (ChIP‐seq) (Lin *et al*., 2010; Ochiai *et al*., 2012; Ouyang *et al*., 2012; Bai *et al*., 2013; Eijkelenboom *et al*., 2013; Kim *et al*., 2013; Riedel *et al*., 2013; Webb *et al*., 2013). These studies have implicated FOXOs in the regulation of target genes related to organismal aging and longevity, including stem cell maintenance, inflammation, oxidative stress, metabolism, and DNA repair (Tran *et al*., 2002; Tothova *et al*., 2007; Paik *et al*., 2009; Renault *et al*., 2009; Ochiai *et al*., 2012; Ouyang *et al*., 2012; Kim *et al*., 2013; Webb *et al*., 2013). However, whether the direct FOXO target genes involved in these processes are specific or conserved across cell types and species has not been determined. A characterization of FOXO direct targets throughout evolution would reveal the extent to which longevity networks are conserved across species, or whether species‐specific networks might impact aging.

Here, we present a meta‐analysis of direct targets of FOXO transcription factors in *C. elegans*,* Drosophila,* mouse, and human based on our own data and publicly available datasets. By designing a uniform pipeline to directly compare FOXO binding datasets, we find that FOXOs bind to both shared and tissue‐specific targets in the mouse. We identify for the first time a relatively large set of direct FOXO targets that are conserved across evolution. We also identify FOXO target genes that change in expression with age as well as potential FOXO cofactors. Together, this study reveals the specificity and conservation of FOXO regulatory networks across tissues and species and suggests mechanisms by which FOXO factors extend lifespan and promote cellular homeostasis.

## Results

### FOXO transcription factors bind shared and tissue‐specific targets in the mouse

**Figure 1:**
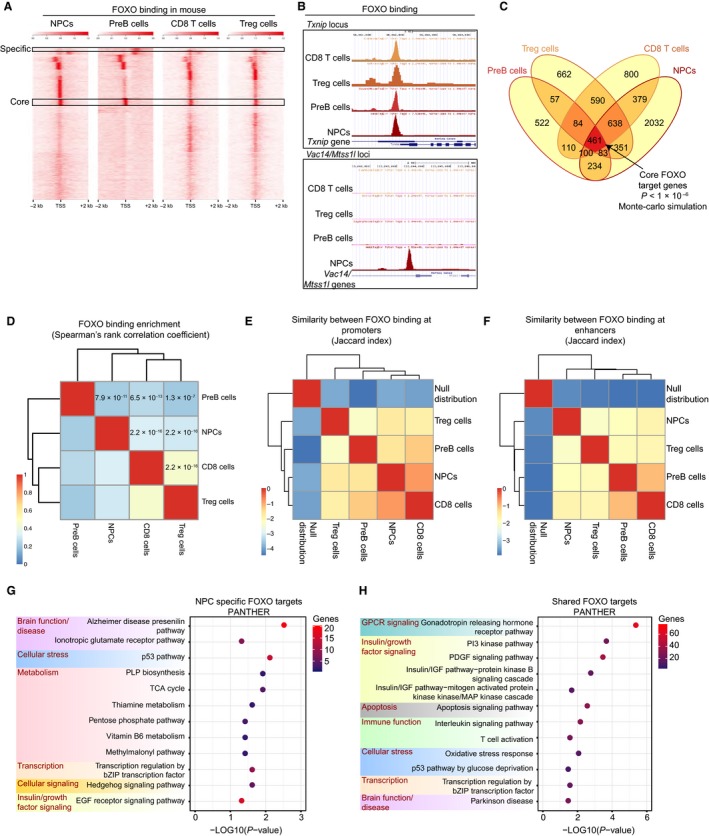
FOXO transcription factors bind tissue‐specific and shared targets in the mouse, and the shared targets are involved in tissue homeostasis and organismal longevity. (A) Enrichment of FOXO binding around transcriptional start sites (TSSs) (±2 kb) in mouse NPCs, PreB cells, CD8+ T cells, and Treg cells. (B) FOXO binding at specific loci in the mouse in all four cell types listed in (A). The upper panel illustrates a common FOXO binding site at the *Txnip* locus across all mouse cell types analyzed. The bottom panel shows NPC‐specific FOXO3 binding at the *Vac‐14/Mtss1 l* loci. (C) Venn diagram depicting gene overlaps in all cell types analyzed. Overlap between all four datasets (dark red) is statistically significant (*P* < 1 × 10^−6^, Monte Carlo simulation; all unique mouse genes were used as background, 19965 genes) and identifies 461 ‘core’ FOXO direct targets in the mouse. (D) Heatmap depicting Spearman's rank correlation (rho) comparing FOXO binding enrichments at genes in the four mouse cell types. Spearman *P*‐values for each comparison are shown. (E) Jaccard statistic representing similarity in FOXO binding at promoters (‐5 kb/+1 kb around TSSs) between cell types and relative to the null distribution. (F) Same as (E), but comparing peaks at enhancers (defined as binding outside of the ‐5 kb/+1 kb promoter window) (G) PANTHER Pathway analysis of the NPC‐specific targets and (H) the 3088 FOXO targets shared among at least two cell types (dark red + orange in (C)).

To investigate whether binding of FOXO transcription factors is mostly cell type‐specific or shared across tissues, we interrogated FOXO ChIP‐seq binding data generated in our laboratory or publicly available from various mouse cell types (Fig. S1A). For direct comparison, we processed the available raw sequencing data using a uniform and stringent pipeline (Fig. S1B). Of the available datasets, endogenous FOXO binding datasets from four different cell types passed our quality control thresholds (Fig. S1A–C and Materials and Methods): FOXO3 binding in neural progenitor cells (NPCs) (Webb *et al*., 2013), FOXO1 binding in T regulatory cells (Treg cells) (Ouyang *et al*., 2012), FOXO1 binding in memory CD8+ T cells (Kim *et al*., 2013), and FOXO1 binding in PreB cells (Ochiai *et al*., 2012). For the purpose of comparison and because there are, as of now, no studies including more than one FOXO isoform, we will not distinguish FOXO isoforms in the remainder of this analysis and we call them all ‘FOXO’. We first examined FOXO binding enrichment around mouse transcription start sites (TSSs) in different cell types using a k‐means clustering analysis (Fig. 1A). We observed that while some FOXO binding is shared across tissue types, some binding is cell type specific (Fig. 1A and B). Consistent with this observation, comparison of the lists of genes bound by FOXO in each cell type confirmed that many FOXO‐bound genes are cell type specific (522 genes in PreB cells, 662 genes in Treg cells, 800 genes in CD8+ T cells, and 2032 genes in NPCs) (Fig. 1C and Table S1). On the other hand, many FOXO‐bound genes, 3088 (43.5% of all targets), were found in at least two cell types, representing the ‘shared FOXO direct targets’ (Fig. 1C and Table S1). Shared targets included a number of previously identified FOXO target genes (e.g., *Cdkn1b/p27*,* Cited2,* and *Txnip*) (Medema *et al*., 2000; Bakker *et al*., 2007; de Candia *et al*., 2008) as well as novel targets (e.g., *Sqstm1*,* Creb1*,* Ezh1,* and *Tbx6*). Interestingly, a significant number of genes, 461 (6.5%, *P *< 1 × 10^−6^, Monte Carlo simulation), are bound by FOXO in all cell types, representing the ‘core FOXO’ direct targets (Fig. 1C and Table S1). FOXO binding also occurs at genes that are not expressed, which could skew the global analysis. Therefore, we also restricted the overlap to genes expressed in each tissue and found that the overlap between the four datasets was still statistically significant (10,197 expressed genes; *P* < 1 × 10^−6^, Monte Carlo simulation) under these more restrictive conditions. To independently examine the correlation between FOXO binding enrichments at target genes in each cell type, we used Spearman's rank correlation. There was a significant correlation between the datasets (Fig. 1D), supporting the notion that FOXO transcription factors share common targets in different mouse tissues.

FOXOs bind their targets at both promoters and enhancers (Eijkelenboom *et al*., 2013; Webb *et al*., 2013). We thus assessed the extent to which FOXO binding was similar at promoter and enhancer regions in different mouse cell types. To do so, we calculated the Jaccard index (an index of similarity, see Materials and Methods) between each cell type at promoters (5 kb upstream/1 kb downstream of TSSs) and more distal binding elements. In both cases, there was strong similarity between the datasets relative to the null distribution (Fig. 1E–F). As a positive control, we calculated the Jaccard index comparing FOXO3 binding in NPCs and the promoter mark trimethylated lysine 4 on histone H3 (H3K4me3) at promoters or the enhancer mark monomethylated lysine 4 on histone H3 (H3K4me1) at distal regions. These Jaccard indexes fall within the range of values observed comparing FOXO binding across cell types (−1.79 for H3K4me3 and FOXO3 at promoters and −1.84 for H3K4me1 and FOXO3 at enhancer regions, compare to Fig. 1E–F). Thus, the similarity between FOXO binding across mouse cell types occurs both at promoters and enhancers.

We then asked whether FOXO targets had different functions depending on whether they were cell type specific or shared, using the Protein Analysis Through Evolutionary Relationships database (PANTHER) (Mi *et al*., 2013). Cell type‐specific FOXO‐bound genes had pathway signatures that were consistent with the tissue of origin, including chemokine and cytokine signaling in immune cell types and Alzheimer's disease–presenilin pathway in NPCs (Figs 1G and S2A–C). Rank‐based gene set enrichment analysis (GSEA) of FOXO targets in each tissue also confirmed hallmark signatures in each cell type, including immune signaling for PreB cells and T cells (Fig. S3A–D). In contrast, PANTHER analysis on FOXO‐bound genes shared by at least two cell types (all orange and red area in Fig. 1C) and shared by all four cell types (‘core’) (red area in Fig. 1C) revealed that that these genes were most enriched for G‐protein‐coupled receptor signaling (GPCR), insulin/growth factor signaling, apoptosis, p53 signaling, and oxidative stress and metabolism (Figs 1H and S2D–E), consistent with previous work on individual genes or gene expression datasets (Brunet *et al*., 1999; Kops *et al*., 2002; Salih & Brunet, 2008; Renault *et al*., 2011). FOXO shared targets were also bound to genes involved in pathways that were not previously known to be regulated by FOXO (interleukin signaling, transcriptional regulation by bZIP factors; Fig. 1H). Core FOXO targets (red in Fig. 1C) were enriched for transcriptional regulation (chromatin organization and regulation by RNA Pol II) and metabolism (Fig. S2E). This finding is consistent with FOXO's ability to open compacted chromatin (Zaret & Carroll, 2011), interact with chromatin remodeling factors (Riedel *et al*., 2013), and regulate glucose metabolism (Puigserver *et al*., 2003; Matsumoto *et al*., 2007). There were no significant PANTHER pathways in core FOXO‐bound genes, possibly because of the small number of genes (461) in this analysis. Collectively, these findings suggest that FOXOs not only perform specialized cell type‐specific function, but also regulate common processes across cell types.

To test whether the FOXO targets (bound in any cell type) included pathways and processes involved in aging or longevity, we overlapped the mouse FOXO targets with aging‐related genes from the GenAge database (Tacutu *et al*., 2013). STRING network analysis was used to display the data (Franceschini *et al*., 2013). There were 53 FOXO‐bound genes that are known to functionally impact aging in the mouse. Interestingly, the functional aging network bound by FOXO included three aging subnetworks: growth hormone/insulin/IGF/TOR signaling (e.g., *Gh*,* Igf1r*,* Akt1,* and *Irs2*), redox regulation/oxidative stress (e.g., *Cat*,* Txn1,* and *Prdx1*), and p53/DNA damage repair (*Ercc2*,* Atr,* and *Trp53*) (Fig. S4). Shared and core FOXO‐bound genes were also enriched for pathways involved in several additional aging‐related processes, including chromatin regulation, immune function, and neurodegenerative disease (Parkinson's disease in the shared FOXO targets and Alzheimer's disease in NPC‐specific targets) (Figs 1H and S2E). Closer examination of the genes in these pathways revealed a number of targets involved in proteostasis (e.g., the proteasome subunit *Psma6*, the heat shock factor *Hspa1b,* and the E3 ubiquitin protein ligase *Fbxw7*). These results indicate that mammalian FOXO targets involve a large number of aging‐related genes. Notably, FOXOs bind both ‘pro‐longevity’ and ‘antilongevity’ GenAge functional regulators of aging. Thus, while FOXO is a central hub in regulating a network of genes involved in aging, some targets may actually antagonize FOXO's role in longevity.

### Conservation of FOXO binding from *Caenorhabditis elegans* to mouse and functional relevance of the conserved FOXO targets

The enrichment for aging‐related genes in FOXO targets in the mouse raises the question of whether the mammalian FOXO targets include components from an ancestral network that regulates aging. In *C. elegans*, FOXO/DAF‐16 is normally inhibited by signaling through the DAF‐2 insulin receptor. In *daf‐2* long‐lived mutants, FOXO/DAF‐16 is localized primarily in the nucleus, and FOXO/DAF‐16 target genes are transcribed (Murphy *et al*., 2003; Oh *et al*., 2006). We first reanalyzed the publicly available raw ChIP‐seq data from whole young adult worms generated by modENCODE and (Riedel *et al*., 2013) (Fig. S1A–C) using our pipeline. Most FOXO/DAF‐16‐bound targets identified in the long‐lived *daf‐2* mutant were also bound in wild‐type worms (3801/4478; 84.9%; *P* < 2.2 × 10^−306^ in Fisher's exact test) (Fig. S5A–C and Table S2). There was significant overlap between FOXO/DAF‐16 targets identified by ChIP‐seq and those identified by DamID (Schuster *et al*., 2010) (443 targets; 48.7%; *P* = 4.76 × 10^−238^ in Fisher's exact test) (Fig. S5D). PANTHER analysis showed that FOXO/DAF‐16 direct targets are enriched for genes in the ubiquitin–proteasome pathway, growth factor signaling pathways, and processes such as metabolism (Figs S5E–F and S6A–B), as previously shown (Murphy *et al*., 2003; Oh *et al*., 2006; Schuster *et al*., 2010; Vilchez *et al*., 2012). In addition, PANTHER analysis revealed enrichment for other aging‐related processes such as immune function, aging‐related diseases, and cell cycle (Fig. S5E–F). A number FOXO/DAF‐16 targets are functional regulators of lifespan in GenAge (419 genes), and these aging‐related genes are clustered into three subnetworks: Insulin/IGF/TOR signaling, translation/ribosome subunits, and metabolism/mitochondrial function (e.g., *atp‐5*,* cco‐1,* and *nuo‐2*) (Fig. S7). This reanalysis of published data confirms and extends previous findings, and serves as a foundation to probe the evolution of the shared FOXO network between invertebrates and mammals.

**Figure 2:**
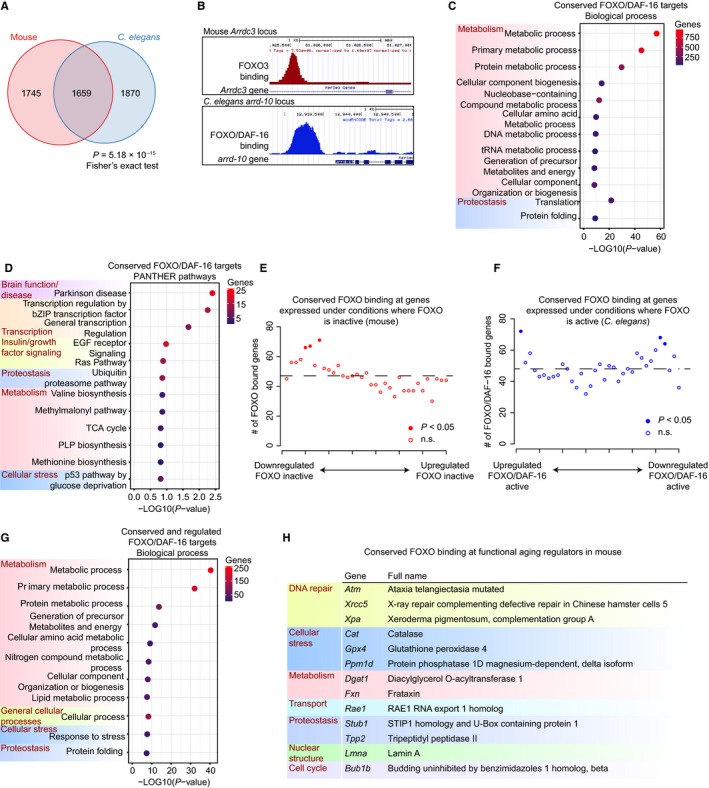
FOXO binding is conserved between *Caenorhabditis elegans* and mouse, and conserved targets are transcriptionally regulated and function in cellular maintenance and repair. (A) Overlap between FOXO target genes in all mouse tissues and FOXO/DAF‐16 target genes (union of wild‐type and *daf‐2* mutants) in *C. elegans* (*P* = 5.18 × 10^−15^, Fisher's exact test; all unique mouse–*C. elegans* orthologs were used as background; 8072 genes). (B) An example of conserved FOXO/DAF‐16 at a specific target, *Arrdc3/arrd‐10*, in mouse and *C. elegans*. (C) PANTHER Biological Process and (D) PANTHER Pathway analysis of conserved FOXO/DAF‐16 target genes. (E) Enrichment for conserved FOXO/DAF‐16 binding sites (overlap in (A)) among genes downregulated in *Foxo1* knockout Treg cells compared to wild‐type. Genes are ranked (X‐axis) by fold change and binned into 35 bins, with genes most downregulated in *Foxo1* ^−/−^ on the left. The number of conserved FOXO targets was calculated for each bin and plotted on the Y‐axis. Filled circles depict statistically significant enrichment in direct binding (*P* < 0.05, Monte Carlo simulation with Benjamini–Hochberg correction). (F) Same analysis as described in (E), but enrichment is shown for targets upregulated by FOXO/DAF‐16 (left) or downregulated by FOXO/DAF‐16 (right). (G) PANTHER Biological Process analysis of mouse/worm conserved targets that are also activated by FOXO. (H) The 13 conserved FOXO/DAF‐16 targets are known to functionally regulate aging in the mouse (GenAge database).

To determine whether direct FOXO binding is conserved from *C. elegans* to mouse, we performed a cross‐species analysis using orthologs (see Materials and Methods). Interestingly, we observed significant overlap between FOXO targets in mouse (bound in any tissue) and FOXO/DAF‐16‐bound genes in *C. elegans* (bound in wild‐type or *daf‐2*) (*P* = 5.18 × 10^−15^ in Fisher's exact test; Fig. 2A–B and Table S3). Moreover, FOXOs tend to bind genes that are conserved from *C. elegans* to mouse more than genes that are species‐specific (mouse genes without orthologs in *C. elegans*) (*P* = 1.06 × 10^−97^ in Fisher's exact test). Together, these results indicate target conservation for FOXO transcription factors across species. As *C. elegans* and mouse diverged at least 500 million years ago, this result also raises the possibility that the FOXO network in mammals and worms is very similar to the ancient regulatory network in their common ancestor.

What is the main function of the evolutionarily conserved FOXO/DAF‐16 targets? We first tested whether the 1659 direct FOXO/DAF‐16 targets conserved between *C. elegans* and mouse (Fig. 2A) were enriched for particular processes or pathways. PANTHER analysis again revealed that growth factor signaling and metabolism were highly enriched among the conserved FOXO direct targets (Fig. 2C–D). Intriguingly, the most highly enriched pathway among the conserved genes was Parkinson's disease (22 genes; Fig. 2D). This generally annotated Parkinson pathway in fact comprises two main cellular processes: proteostasis (e.g., Hsp70 family members *Hspa1a* and *Hspa14*, proteasome subunits *Psma2* and *Psmb1*, and the E3 ubiquitin protein ligase *Park2*), and cell cycle (e.g., the G1/S regulator *Ccne1*). This analysis also uncovered novel pathways downstream of FOXO/DAF‐16 such as transcriptional regulation by bZIP transcription factors (14 genes) and tRNA metabolic processes (26 genes, *P* = 1.68 × 10^−10^) (Fig. 2C–D). This latter category includes tRNA synthases and ligases that impinge on tRNA levels and translation. Among genes that functionally regulate lifespan in *C. elegans* and mouse (cataloged in the GenAge database), FOXO/DAF‐16 targets conserved between *C. elegans* and mouse were enriched for DNA repair, regulation of oxidative stress, insulin/IGF/TOR signaling, translational regulation, and metabolism (Figs 2H and S8). Thus, FOXO/DAF‐16 functions as a conserved hub that affects aging and lifespan via an integrated network of aging‐related processes.

Transcription factor binding in the absence of regulation has been observed for several transcription factors, including FOXO/DAF‐16 (Schuster *et al*., 2010; Bai *et al*., 2013). Therefore, we next asked whether the conserved FOXO/DAF‐16 targets are transcriptionally regulated by these factors. We used a meta‐analysis of FOXO/DAF‐16 gene expression datasets (Tepper *et al*., 2013) as well as microarray expression datasets from mouse cell types where ChIP‐seq has been performed. Taking advantage of existing gene expression datasets in the mouse, we ranked all genes by fold change comparing wild‐type to FOXO knockout conditions. We found that the conserved FOXO/DAF‐16 direct targets were enriched among the genes most downregulated upon knockout of *Foxo1* in Treg cells (Ouyang *et al*., 2012) (Fig. 2E). By interrogating the existing meta‐analysis of DAF‐16‐transcribed genes in *C. elegans* (Tepper *et al*., 2013) (comparing *daf‐2* mutants vs. *daf‐2;daf16* mutants), we observed that DAF‐16 binding is enriched at the genes most upregulated in conditions that activate DAF‐16 (Fig. 2F). A PANTHER gene ontology analysis of the FOXO/DAF‐16 targets conserved between *C. elegans* and mouse, and regulated in the expression analysis targets, revealed enrichment for genes involved in metabolism, cellular stress, and proteostasis (Fig. 2G). Together, these data suggest that conserved FOXO/DAF‐16 target genes are indeed transcriptionally regulated by FOXO in both mice and worms, but that, consistent with previous findings (Schuster *et al*., 2010; Bai *et al*., 2013), FOXO binding was also observed at genes that were not transcriptionally regulated. An interesting possibility is that FOXOs may regulate the latter targets in response to specific environmental conditions, such as stress stimuli.

### FOXO targets conserved in *Caenorhabditis elegans*,* Drosophila*, mouse, and human

**Figure 3:**
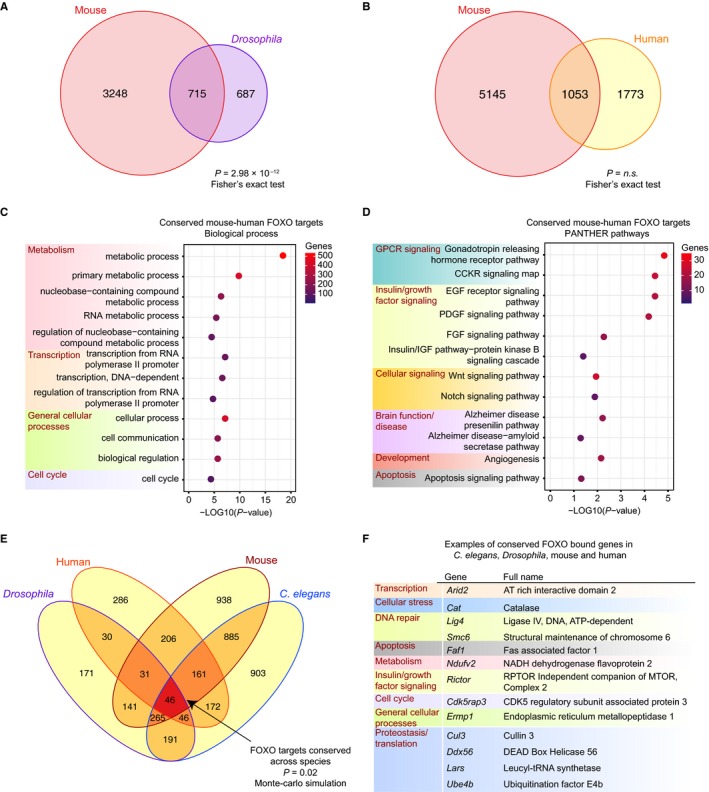
Conservation of binding between *Drosophila* dFOXO and mouse FOXO, human FOXO and mouse FOXO, and among all species examined. (A) Venn diagram representing overlap between direct binding by dFOXO in *Drosophila* and mouse FOXO (*P* = 2.98 × 10^−12^, Fisher's exact test; all unique mouse–*Drosophila* orthologs were used as background; 9314 genes). (B) Venn diagram representing overlap between direct binding by FOXO in mouse and human FOXO (*P* = not significant, Fisher's exact test; all unique mouse–human orthologs were used as background; 16754 genes). (C) PANTHER Biological Process and (D) PANTHER Pathway analysis of the 1053 FOXO direct target genes conserved between mouse and human. (E) Overlap among FOXO targets in *Caenorhabditis elegans*,* Drosophila*, mouse, and human. The overlap (dark red) is statistically significant (*P* = 0.02, Monte Carlo simulation; all genes with orthologs across these species were used as background, 5706 genes). (F) Examples of conserved FOXO‐bound genes in *C. elegans*,* Drosophila*, mouse, and human.

We next asked whether FOXO targets are also conserved in other species. Increased activity of the *Drosophila* ortholog of FOXO, dFOXO, prolongs lifespan (Clancy *et al*., 2001; Tatar *et al*., 2001). In addition, SNPs in human *FOXO3* have been associated with longevity. We first compared the binding of FOXO in all four mouse tissues analyzed here to dFOXO binding in *Drosophila* (Alic *et al*., 2011; Bai *et al*., 2013). As dFOXO ChIP‐seq in *Drosophila* resulted in fewer peaks with significant enrichment than expected for ChIP‐seq, we combined the available dFOXO ChIP‐seq and ChIP‐chip data (Fig. S1A–C). We observed that 50.9% of *Drosophila* dFOXO‐bound genes were also bound by mouse FOXO (715 genes, *P* = 2.98 × 10^−12^, Fisher's exact test) (Fig. 3A and Table S4). Functionally, the mouse–*Drosophila* conserved genes closely resembled the mouse–*C. elegans* conserved pathways and processes, including metabolism, growth factor signaling, proteostasis, and transcriptional regulation (Fig. S9A–B). Together, these results highlight the strong cross‐species conservation among FOXO direct target genes and their cellular function.

Similarly, we compared FOXO binding in mouse cells to genome‐wide binding in human cells (Fig. 3B) (Eijkelenboom *et al*., 2013). Although the FOXO ChIP‐seq in human cells did not reach the threshold of our pipeline (due to small fragment length in the ChIP‐seq), it is so far the only available data for FOXO binding in human. Thus, we compared it to our analysis in mouse. We identified 1053 FOXO target genes that are conserved between mouse and human (Fig. 3B and Table S5). Consistent with what we observed from other species, the genes that are conserved between mouse and human are enriched for growth factor signaling, metabolism, transcriptional regulation, and cell cycle regulation (Fig. 3C–D). Thus, functional regulation by FOXO factors appears to be conserved from *C. elegans* through human. It will be important to compare FOXO binding across species as additional human datasets become available to gain additional insight into the conservation and differences of FOXO targets between mouse and human.

Comparing FOXO binding among all species examined showed significant overlap between FOXO targets in *C. elegans*,* Drosophila*, mouse, and human (*P* = 0.02, Monte Carlo simulation). This overlap identified 46 targets conserved across these four species (Fig. 3E and Table S6). These 46 conserved targets (see Fig. 3F for examples and Table S6 for full list) are enriched for growth factor signaling pathways, the p53 pathway, metabolism and proteostasis pathways, and those processes have all been implicated in the aging process (Lopez‐Otin *et al*., 2013). Therefore, FOXO binding is conserved in invertebrates and mammals, and the processes downstream of conserved targets include key regulators of longevity that could have been conserved throughout evolution.

### Expression of some FOXO direct targets, but not the conserved core, changes with age

**Figure 4:**
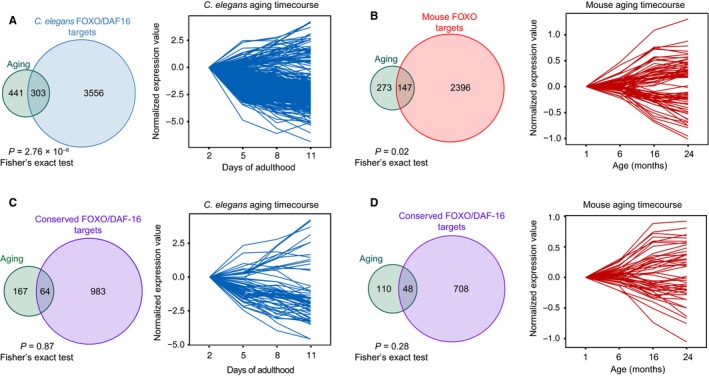
A subset of FOXO targets changes with age. (A) Overlap between *Caenorhabditis elegans* DAF‐16 targets and genes that are either upregulated or downregulated with age in this species. The overlap is shown in the upper left (Venn diagram, *P* = 2.76 × 10^−6^, Fisher's exact test; background is all unique genes on the worm aging microarray, 11706 genes), and the expression profile of the overlapping targets during aging is shown on the right. (B) The same arrangement as in (A) showing comparisons between mouse genes that change with age and FOXO binding in the mouse (*P* = 0.02; Fisher's exact test; background is all unique genes on the mouse aging microarray, 8400 genes). (C‐D) Same as (A), depicting overlap between conserved FOXO/DAF‐16 binding at *C. elegans* genes (*P* = 0.87; Fisher's exact test; background is all unique genes on the *C. elegans* aging microarray and with mouse–*C. elegans* orthologs, 3407 genes) and mouse genes (*P* = 0.28, Fisher's exact test; background is all unique genes on the mouse aging microarray and with mouse–*C. elegans* orthologs, 2694 genes) that change with age.

Are FOXO target genes conserved across species affected, or on the contrary preserved, transcriptionally, during aging? Using aging time course data from DNA microarrays from mouse (brain and thymus) and *C. elegans* (Zahn *et al*., 2007; Budovskaya *et al*., 2008), we first calculated the overlap between genes that change with age and FOXO/DAF‐16 binding in *C. elegans*, FOXO binding in mouse, and conserved FOXO/DAF‐16 targets (Fig. 4A–D, left panels and Table S7). In each case, we identified direct target genes that are either upregulated or downregulated with age (Fig. 4A–D, right panels). The strongest overlap between direct FOXO/DAF‐16 binding and age‐related changes in expression was in *C. elegans* (303 genes, *P* = 2.76 × 10^−6^, Fisher's exact test), although FOXO direct targets in mouse also exhibited change in expression with age (147 genes, *P* = 0.02, Fisher's exact test). While we did identify several conserved FOXO targets that changed with age (Fig. 4C–D) (64 in *C. elegans* and 48 in mouse), the overlap was not statistically significant in either case, suggesting that most genes that change in expression with age are actually not conserved direct targets of FOXO/DAF‐16. These results are consistent with the notion that changes in the highly conserved activity of FOXO transcription factors are not directly responsible for the age‐related changes to global gene expression networks during aging. Instead, the conserved gene expression network downstream of FOXOs may be crucial for preserving cells and tissues even in older animals.

While the majority of targets do not change in expression with age, some FOXO target genes did change, and may therefore be sufficient to underlie tissue deterioration during aging. In *C. elegans*, FOXO/DAF‐16 targets that change in expression with age are clustered into several functional groups: collagen, metabolism (fat, lipid, and glucosamine), Notch signaling, and the heat shock response (Fig. S10). Interestingly, the network signature among the mouse FOXO targets that change with age is different and includes immune function, translation, and cell cycle genes (Fig. S11). Collagen remodeling, lipid and fatty acid metabolism, and Notch have been implicated in aging (Dresen *et al*., 2015; Ewald *et al*., 2015; Liu & Li, 2015). Similarly, in the mouse, translation, immune function, and cell cycle pathways are integral to aging and longevity (Chung *et al*., 2009; Johnson *et al*., 2013; Hofmann *et al*., 2015).

### Conserved cofactors at FOXO targets

**Figure 5:**
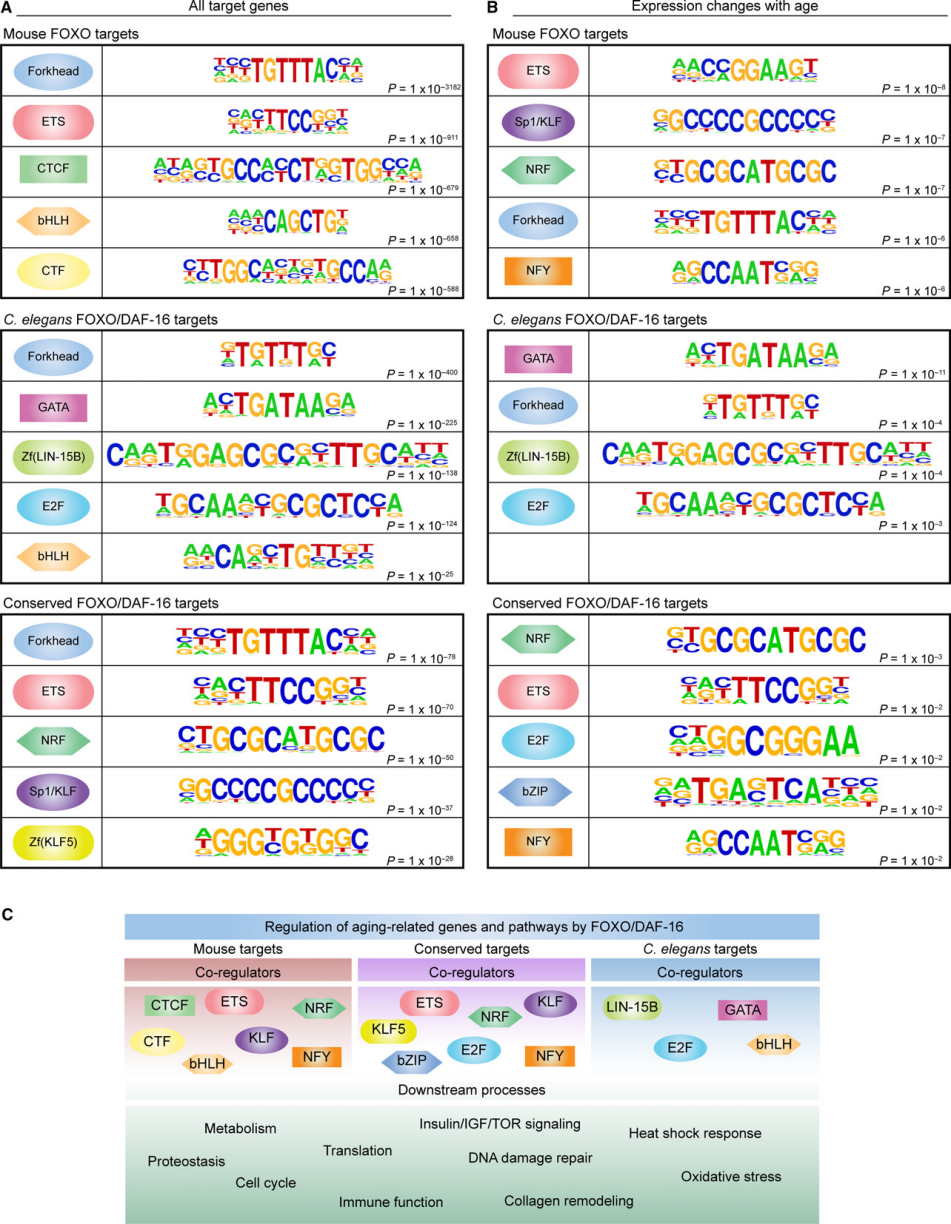
FOXO binding sites are enriched for candidate coregulator motifs. (A) The top five most highly enriched motifs within 200 bp of FOXO in mouse, FOXO/DAF‐16 in *Caenorhabditis elegans*, or conserved FOXO/DAF‐16 targets. (B) The top five ranked motifs within 200 bp of FOXO in mouse, FOXO/DAF‐16 in *C. elegans*, or conserved FOXO/DAF‐16 targets for genes that change with age in the respective species (mouse genes were used in the conserved analysis). Note that only four motifs were enriched in the *C. elegans* FOXO/DAF‐16 group (center). (C) Summary of the candidate FOXO coregulators in mouse, *C. elegans*, and conserved between species, and the downstream processes identified in this study.

Previous studies have implicated other transcription factors as FOXO coregulators, which can function synergistically or antagonistically with FOXOs (Lin *et al*., 2010; Alic *et al*., 2011; Webb *et al*., 2013). However, only a limited number of FOXO cofactors have been identified, and it is unknown whether FOXO/co‐factor interactions are tissue specific and conserved across species. To identify candidate cofactors across tissues and species, we performed *in silico* motif analysis of FOXO binding sites shared among mouse tissues, FOXO/DAF‐16 binding sites in *C. elegans*, and conserved targets (*C. elegans* to mouse). In all cases, the Forkhead consensus motif (TGTTTAC) was the top enriched motif (Fig. 5A) (Furuyama *et al*., 2000). Shared FOXO binding sites in the mouse were also highly enriched in ETS, CTCF, bHLH, and CTF motifs (Fig. 5A). Thus, the same transcription factor family may function together with FOXOs in different mouse tissues. In *C. elegans*, the top five most enriched motifs were different from the mouse motifs, with the exception of the Forkhead and bHLH motifs. In addition, *C. elegans* DAF‐16 binding sites were highly enriched for GATA, LIN‐15B (a zinc finger transcription factor), and E2F motifs. The enrichment for GATA family factors is notable as this family includes ELT factors and PQM‐1, and these factors have been previously implicated in aging in *C. elegans* (Budovskaya *et al*., 2008; Tepper *et al*., 2013; Zhang *et al*., 2013). Similar to mouse FOXO targets, conserved targets were highly enriched for the ETS family motif, and the analysis also revealed motifs for Sp1/KLF family members and NRF factors (Fig. 5A). ETS proteins have been implicated in the regulation of aging in *Drosophila* (Alic *et al*., 2014), NRF factors (nuclear respiratory factors, which are different from SKN‐1/nuclear factor erythroid‐related factor) regulate metabolism and mitochondrial function (Scarpulla, 2002), and KLF factors have been implicated in fat metabolism in *C. elegans* and adipocyte differentiation in mammals (Brey *et al*., 2009; Carrano *et al*., 2014). Thus, this analysis suggests a potential cooperation between these factors and FOXO transcription factors in coregulating target genes and processes that regulate aging and longevity.

Motif analysis on the few FOXO targets that change with age (Fig. 5B) revealed enrichment of motifs for ETS and NRF motifs in mouse, and GATA factors in *C. elegans*. Conserved targets also exhibited enrichment for E2F, bZIP, and NFY motifs. Interestingly, E2F regulates cellular senescence in mammalian cells, and E2F (*efl‐1*) knockout in *C. elegans* extends lifespan in a *daf‐16*‐dependent manner (Xie *et al*., 2014). Intriguingly, the bZIP family of transcription factors includes CREB, which has been implicated in lifespan regulation and loss of learning and memory with age in worms (Kauffman *et al*., 2010; Mair *et al*., 2011). Together, these analyses reveal potential coregulators of FOXO transcription factors, which set the foundation for the discovery of novel transcription factor partnerships in cell type‐specific and organismal functions of FOXO transcription factors.

## Discussion

FOXO transcription factors play a pivotal role in regulating longevity in various species, yet the conservation of specific processes directly downstream of FOXO has not been determined. Our study is the first to compare the direct FOXO targets between species and among mammalian tissues. This approach allowed us to delineate both the shared targets of FOXO and the cell type‐specific targets in mammals. An interesting possibility is that the shared FOXO targets may be the most important for the regulation of lifespan, whereas the tissue‐specific targets may be involved in other functions. Whether the tissue‐specific targets contribute to, or are at odds with, FOXO's role in longevity by regulating tissue homeostasis awaits further investigation. It will be important to understand these tissue‐specific functions as our results suggest that interventions that affect lifespan would have dissimilar effects on different tissues.

In addition to processes that are known to regulate aging downstream of FOXOs, we also observed enrichment for conserved FOXO binding at novel targets. For example, transcription regulation by bZIP transcription factors was enriched among the conserved FOXO targets. This family comprises a number of transcription factors involved in diverse processes, and includes the stimulus‐induced subfamilies CREB (cAMP response element binding protein) and AP‐1 (activator protein 1). CREB has been implicated in the regulation of longevity and tissue function during aging in *C. elegans*, although its role is complex, as it has both pro‐longevity and antilongevity functions (Kauffman *et al*., 2010; Mair *et al*., 2011). Our observation that the bZIP family as a whole is enriched downstream of FOXOs suggests that the bZIP family may include additional undiscovered regulators of longevity.

Only a subset of the FOXO/DAF‐16 targets change in expression with age, consistent with previous studies (Tepper *et al*., 2013). Surprisingly, the FOXO targets that are conserved across species do not tend to change in expression with age. This finding may indicate that the conserved FOXO targets are the most critical for preserving tissues during aging. This result also suggests that changes in gene expression during aging may be mostly species‐ and/or cell type specific.

In summary, our genome‐wide meta‐analysis of the direct FOXO/DAF‐16 targets across mammalian cell types and across different species revealed both cell type‐specific and shared targets. This analysis provides a valuable resource to the aging community, as these findings are consistent with FOXO's position as a central node in the regulation of longevity and suggest a coevolution of FOXO and its network of metabolic and stress resistance targets. Defining both the cell type‐specific and conserved network downstream of FOXOs should help identify ways to delay aging and age‐related diseases.

## Materials and methods

### List of raw datasets used

ChIP‐seq datasets: GSE48336 (mouse NPCs), GSE35024 (mouse PreB cells), GSE46944 (mouse CD8+ T cells), GSE40657 (mouse Treg cells), GSE15567 (*C. elegans daf‐2* mutants), GSE15567 (*C. elegans* wild‐type), GSE35486 (human DLD‐1 cells), GSE44686 (*Drosophila chico* heterozygotes and adults with ablated IPCs).

Microarray data: GSE18326 (mouse NPCs), GSE10273 (mouse PreB cells), GSE46942 (mouse CD8+ T cells), GSE40655 (mouse Treg cells).

### Processing of ChIP‐seq data

Raw sequencing reads (FASTQ files) for the ChIP‐seq datasets (see Fig. S1) were downloaded from the GEO database and processed with the following pipeline; reads were mapped to the appropriate genome using Bowtie 1 (v1.0.0 http://bowtie-bio.sourceforge.net/index.shtml; mm9, ce10, dm3, or hg19) (Langmead *et al*., 2009), and duplicate reads were marked with Picard and removed with SAMtools. Library quality was assessed based on the ENCODE quality control metrics (https://www.encodeproject.org/data-standards/2012-quality-metrics/). Briefly, normalized strand and relative strand cross‐correlation scores (NSC and RSC) were calculated (Marinov *et al*., 2014). Mammalian libraries with NSC > 1.1 and RSC > 1 and invertebrate libraries with NSC > 1 and RSC > 0.9 passed quality control and were processed for peak calling. Peak calling was performed using MACS 2.0 (v2.0.8 http://liulab.dfci.harvard.edu/MACS/) (Zhang *et al*., 2008). The MACS command was as follows: macs2 ‐t ChIP.bam ‐c Input.bam ‐n OutputName ‐g ce/dm/mm/hs ‐q 0.01–0.0001. As the number of ChIP‐seq peaks varies substantially depending on the antibody, the MACS q‐value was adjusted to obtain similar numbers of peaks, even among high quality libraries. See Fig. S1 for MACS q‐values used for each library. For mouse datasets, peaks were assigned to genes using GREAT (v2.0.2, UCSC mm9 annotations, http://bejerano.stanford.edu/great/public/html/) (McLean *et al*., 2010), limiting the peak‐calling window to −5 kb/+1 kb around transcription start sites (TSSs). For the human dataset, the peak‐calling window was the same as for the mouse, but also extended to the nearest gene by 1000 kb as this dataset was previously reported to be enriched for enhancer binding (Eijkelenboom *et al*., 2013). For *Drosophila* and *C. elegans* experiments, peaks were assigned to genes using a −1 kb/+500 bp winding around TSSs (this narrow window prevents false positives in the peak calling), using the dm3 and ce10 Refseq annotations, respectively. *C. elegans ‐* and *Drosophila‐*associated *g*ene names were obtained from BioMart. Binding enrichment around TSSs was performed using NGS plot (https://code.google.com/p/ngsplot/) using the k‐means clustering argument: ngs.plot.r –G mm9 –R tss –E UnionGeneList –GO km –KNC NumberOfClusters –C ConfigurationFile –O OutputName (Shen *et al*., 2014).

### Jaccard analysis

Jaccard indices were calculated using the bedtools v2.22.1 Jaccard function (Favorov *et al*., 2012). To generate the null distribution, datasets were shuffled 10,000 times using the bedtools shuffleBed function. Jaccard values from the 10,000 trials were averaged for representation on the heatmaps. To provide reference values for overlap with enhancers and promoters, Jaccard values for the proximal and distal FOXO3 NPC peaks were generated using the published ChIP‐seq datasets for the enhancer mark H3K4me1 and the promoter mark H3K4me3 in NPCs, respectively (Webb *et al*., 2013). Clustered heatmaps were generated in R using the package Pheatmap v0.7.7 from the CRAN repository.

### Network analysis

Network analysis was performed using STRING (v10) (Szklarczyk *et al*., 2015) and displayed using confidence view.

### Functional annotation

Functional annotation of gene sets was performed using the PANTHER database (http://pantherdb.org/) (Mi *et al*., 2013). Gene lists were uploaded in Gene Symbol format, and Gene Symbol lists (all genes) from the appropriate species were used as background. The Benjamini–Hochberg procedure (Benjamini & Hochberg, 1995) was used for multiple hypothesis correction, and overrepresented pathways were plotted in R using ‘ggplot2’ and implementing the ‘geom_point’ function (http://ggplot2.org/).

### Gene set enrichment analysis

GSEA was performed using the GSEA Preranked option. For input datasets, target genes were ranked by MACS fold enrichment values. The C2: curated gene set collection from the Molecular Signatures Database was used for the analysis.

### Ortholog analysis

*Caenorhabditis elegans* and *Drosophila* orthologs to mouse genes were obtained using the modENCODE project orthologs (http://compbio.mit.edu/modencode/orthologs/). Human–mouse orthologs were obtained from the Mouse Genome Informatics (MGI) Vertebrate Homology Database (http://www.informatics.jax.org/homology.shtml). Cross‐species comparisons were performed using one‐to‐one mouse– *C. elegans*, mouse–*Drosophila,* and mouse–human relationships.

### Motif analysis

Motif analysis was performed using the Homer findGenomeMotifs.pl tool (http://homer.salk.edu/homer/index.html) (Heinz *et al*., 2010). An initial motif analysis was performed using all peaks from each processed dataset (Fig. S1). Additional motif analyses were performed on the union of the mouse or worm peaks, peaks at mouse–*C. elegans* conserved targets, and peaks associated with genes that change in expression with age. Default settings (200 base pair window) were used in all analyses, and *P*‐values from the Homer Known Motif Enrichment Results are reported. In cases where a motif appeared multiple times, only the top (smallest) *P*‐value is reported.

### Venn diagrams and statistical analysis of overlaps

Venn diagrams were created in R using the ‘Vennerable’ package (https://r-forge.r-project.org/projects/vennerable/). Statistical analysis for the significance of overlaps was performed using Fisher's exact test. When calculating significance, we limited the ‘background dataset’ to only the relevant background genes. For the calculation within a species (among the mouse FOXO datasets, and between *C. elegans* datasets), all unique genes within the species were used as ‘background’. For the cross‐species analysis, only the genes with orthologs (mouse–*C. elegans*, mouse–*Drosophila,* or mouse–human orthologs) were used as ‘background’. For the aging analysis, the genes included as probes in the microarrays were used as ‘background’, and the genes on the microarray and with orthologs (mouse–*C. elegans*) for the comparisons of the conserved genes.

### Microarray analysis

Microarrays (Affymetrix) were analyzed using RMA (robust multi‐array analysis) from the ‘affy’ package and the RankProd implementation of the Rank Products method ‘RankProd’ package (Breitling *et al*., 2004) to identify differentially expressed genes. To determine the expressed genes in each mouse cell type, the signal distribution was plotted using the plotDensity function in affy, and an intensity of 6 was used as a threshold for expression. For overlaps between FOXO binding and differential expression, genes were ranked by fold change (*Foxo1* ^*+/+*^ vs *Foxo1* ^−*/*−^ for mouse data) or FOXO/DAF‐16 responsiveness in *C. elegans* (Tepper *et al*., 2013). Genes were binned into 35 bins (similar to (Tepper *et al*., 2013)) and the number of FOXO/DAF‐16‐bound genes was calculated for each bin. Statistical significance between ChIP‐seq binding data and microarray data was performed using 100,000 Monte Carlo simulations, and the *P*‐values were evaluated as 1‐(percentile in simulations), followed by Benjamini–Hochberg correction. ggplot2 was used to plot aging time course expression data.

### Code

The code will be released at: http://www.thewebblab.com/.

## Funding

This work was supported by NIH P01 AG036695 to AB, an Alfred Sloan Foundation Fellowship to AK, and NIH/NIGMS RI‐INBRE Program in Neuroscience P20 GM103430 to AW.

## Conflict of interest

The authors declare no competing financial interests.

## Author contributions

AW, AK, and AB conceived and designed the study. All analyses were performed by AW. AK provided guidance on bioinformatics analysis and established the initial pipeline for processing ChIP‐seq data. The manuscript was prepared by AW and AB.

## Supporting information


**Fig. S1** ChIP‐seq libraries available for meta‐analysis and data processing pipeline.
**Fig. S2** PANTHER Pathway analysis of cell type specific FOXO targets.
**Fig. S3** GSEA analysis of FOXO targets in different mouse cell types.
**Fig. S4** STRING network analysis of mouse FOXO targets that functionally regulate aging.
**Fig. S5** Direct targets of FOXO/DAF‐16 in *Caenorhabditis elegans* are involved in cell signaling, metabolism, and quality control.
**Fig. S6** PANTHER analysis of FOXO/DAF‐16 targets bound in *daf‐2* mutants, but not in wild type in *Caenorhabditis elegans*.
**Fig. S7** STRING network analysis of FOXO/DAF‐16 targets in *Caenorhabditis elegans* that functionally regulate aging.
**Fig. S8** STRING network of FOXO targets conserved between *Caenorhabditis elegans* and mouse that functionally regulate aging.
**Fig. S9** PANTHER analysis of targets conserved between *Drosophila* dFOXO and mouse FOXO.
**Fig. S10** STRING network of FOXO/DAF‐16 target genes in *Caenorhabditis elegans* that change in expression with age in this species.
**Fig. S11** STRING network of FOXO targets in the mouse that change in expression with age in this species.

**Table S1** Mouse FOXO target genes in the four cell types analyzed (NPCs, PreB cells, Treg cells, or CD8+ T cells), cell type‐specific targets, targets that are shared between at least two cell types, or bound in all cell types (Core).

**Table S2** FOXO/DAF‐16 target genes in *Caenorhabditis elegans* (wild‐type, *daf‐2* mutants, and the overlap between wild‐type and *daf‐2* mutants).

**Table S3** FOXO/DAF‐16 target genes that are mouse specific, *Caenorhabditis elegans* specific, or conserved between *C. elegans* and mouse.

**Table S4** FOXO/dFOXO targets that are mouse specific, *Drosophila* specific, or conserved between mouse and *Drosophila*.

**Table S5** FOXO targets that are mouse specific, human specific (mouse names listed), or conserved between mouse and human.

**Table S6** FOXO targets that are conserved across all species (*Caenorhabditis elegans*,* Drosophila*, mouse, and human).

**Table S7** FOXO targets that change in expression with age in the mouse and *Caenorhabditis elegans*.
