# Supplementary material for: Characterization of the direct targets of FOXO transcription factors throughout evolution
Source: Aging Cell. 2016 Apr 8;15(4):673–85. doi: 10.1111/acel.12479 (PMC4933671; doi:10.1111/acel.12479)
Supplement: Supplementary file 1 — Fig. S1 ChIP‐seq libraries available for meta‐analysis and data processing pipeline. Fig. S2 PANTHER Pathway analysis of cell type specific FOXO targets. Fig. S3 GSEA analysis of FOXO targets in different mouse cell types. Fig. S4 STRING network analysis of mouse FOXO targets that functionally regulate aging. Fig. S5 Direct targets of FOXO/DAF‐16 in Caenorhabditis elegans are involved in cell signaling, metabolism, and quality control. Fig. S6 PANTHER analysis of FOXO/DAF‐16 targets bound in daf‐2 mutants, but not in wild type in Caenorhabditis elegans. Fig. S7 STRING network analysis of FOXO/DAF‐16 targets in Caenorhabditis elegans that functionally regulate aging. Fig. S8 STRING network of FOXO targets conserved between Caenorhabditis elegans and mouse that functionally regulate aging. Fig. S9 PANTHER analysis of targets conserved between Drosophila dFOXO and mouse FOXO. Fig. S10 STRING network of FOXO/DAF‐16 target genes in Caenorhabditis elegans that change in expression with age in this species. Fig. S11 STRING network of FOXO targets in the mouse that change in expression with age in this species. [file ACEL-15-673-s001.pdf]

Figure S1

A

Reference	PMID	Factor	Species	Cell type	Accession	Mapped Reads	Passed QC?
Webb et al.	23891001	FOXO3	Mouse	Adult NPCs	GSE48336	12,702,983	Yes
Ochiai et al.	22267219	FOXO1	Mouse	*IRF-4,8$^{-/-}$* pre-B cells	GSE35024	45,382,078	Yes
Kim et al.	23932570	FOXO1	Mouse	CD8+ T-cells	GSE46944	9,949,662	Yes
Ouyang et al.	23135404	FOXO1	Mouse	nTreg	GSE40657	16,328,867	Yes
Litvak et al.	22982991	FOXO3	Mouse	Unstimulated macrophages	GSE37052	5,694,391	No
Lin et al.	20543837	FOXO1	Mouse	Rag1 KO pro-B cells	GSE21978	16,392,076	No
Riedel et al.	23604319	DAF-16::GFP	*C. elegans*	Whole worm	GSE15567	19,379,002	Yes
modEncode	-	DAF-16::GFP	*C. elegans*	Whole worm	GSE15567	3,123,508	Yes
Bai et al.	24244197	dFOXO	*Drosophila*	*chico* heterozygotes	GSE44686	35,627,907	Yes
Bai et al.	24244197	dFOXO	*Drosophila*	Adults with ablated IPCs	GSE44686	35,690,140	Yes
Eijkelenboom et al.	24360957	FOXO3-ER	Human	DLD1 cells	GSE35486	13,302,980	No

B

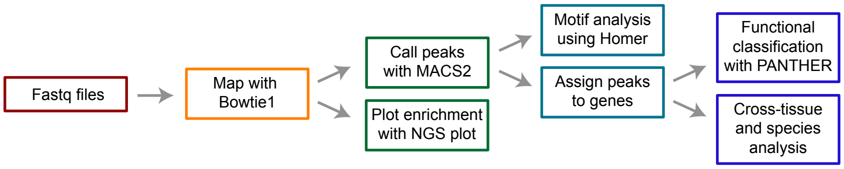

C

Cell type	Factor	MACS peaks	MACS q-value	Target genes	Motif	% Targets with motif	p-value
NPCs	FOXO3	22132	0.001	4278	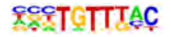	36.12	1×10^{-3101}
CD8+ T-cells	FOXO1	8224	0.1	3162	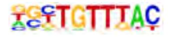	31.34	1×10^{-485}
pre-B cells	FOXO1	14020	0.001	1651	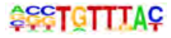	42.48	1×10^{-1909}
Treg cells	FOXO1	6209	0.1	2926	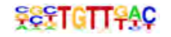	31.63	1×10^{-404}
C. elegans Wild type	DAF-16::GFP	11711	0.001	7189	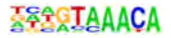	54.89	1×10^{-611}
C. elegans daf-2	DAF-16::GFP	6434	0.1	4478	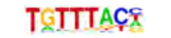	36.73	1×10^{-194}
Drosophila chico heterozygotes	dFOXO	169	0.1	151	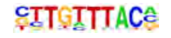	14.20	1×10^{-18}
Drosophila IPC ablation	dFOXO	203	0.1	251	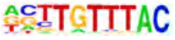	46.31	1×10^{-87}
Human DLD1 cells	FOXO3-ER	2850	0.01	3092	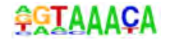	63.72	1×10^{-450}

Figure S2

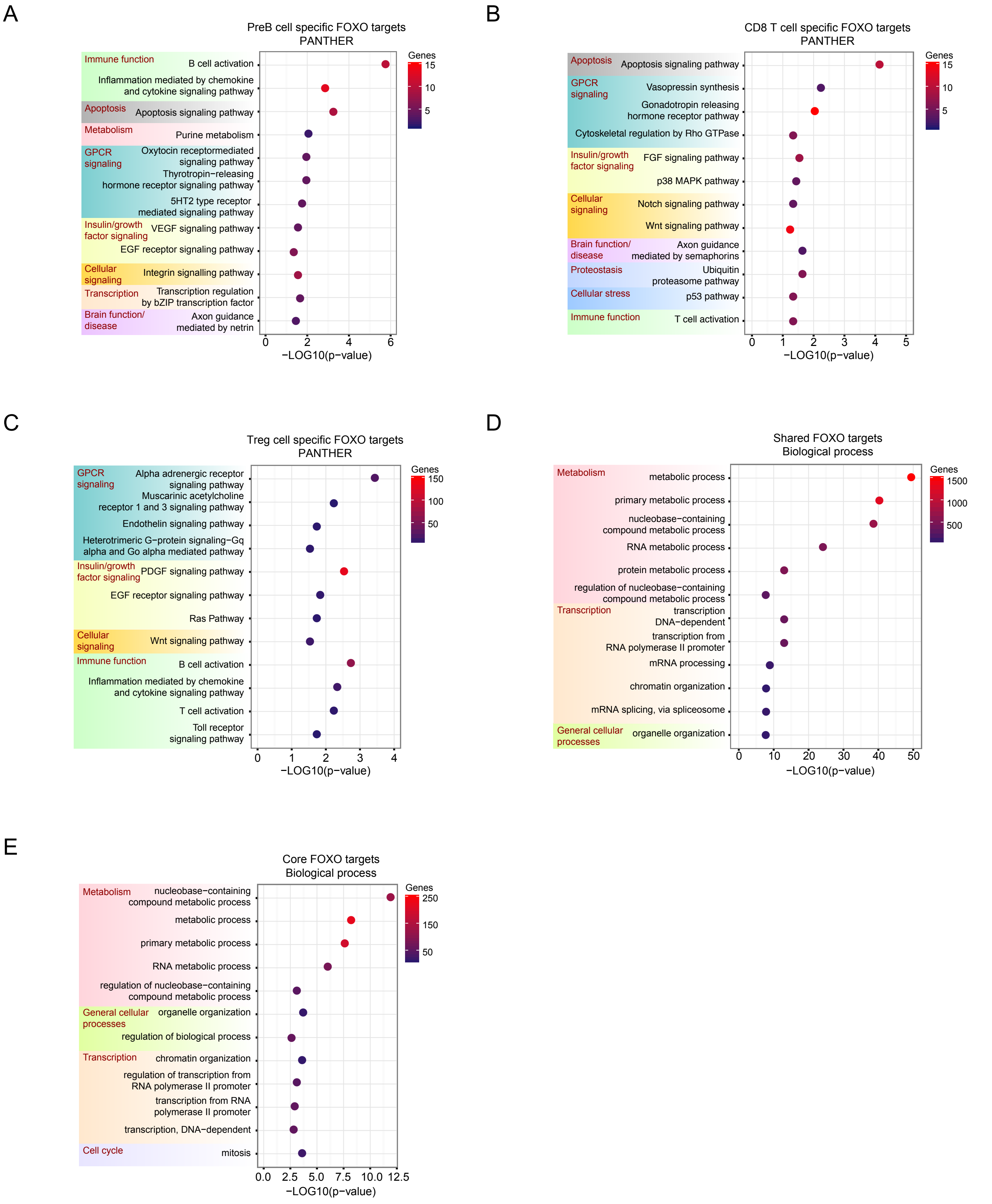

A
PreB cell specific FOXO targets
PANTHER
Immune function
B cell activation
Inflammation mediated by chemokine and cytokine signaling pathway
Apoptosis
Apoptosis signaling pathway
Metabolism
Purine metabolism
GPCR signaling
Oxytocin receptormediated signaling pathway
Thyrotropin−releasing hormone receptor signaling pathway
5HT2 type receptor mediated signaling pathway
Insulin/growth factor signaling
VEGF signaling pathway
EGF receptor signaling pathway
Cellular signaling
Integrin signalling pathway
Transcription
Transcription regulation by bZIP transcription factor
Brain function/ disease
Axon guidance mediated by netrin
0 2 4 6
−LOG10(p−value)
Genes
15
10
5
B
CD8 T cell specific FOXO targets
PANTHER
Apoptosis
Apoptosis signaling pathway
GPCR signaling
Vasopressin synthesis
Gonadotropin releasing hormone receptor pathway
Cytoskeletal regulation by Rho GTPase
Insulin/growth factor signaling
FGF signaling pathway
p38 MAPK pathway
Cellular signaling
Notch signaling pathway
Wnt signaling pathway
Brain function/ disease
Axon guidance mediated by semaphorins
Proteostasis
Ubiquitin proteasome pathway
Cellular stress
p53 pathway
Immune function
T cell activation
0 1 2 3 4 5
−LOG10(p−value)
Genes
15
10
5
C
Treg cell specific FOXO targets
PANTHER
GPCR signaling
Alpha adrenergic receptor signaling pathway
Muscarinic acetylcholine receptor 1 and 3 signaling pathway
Endothelin signaling pathway
Heterotrimeric G−protein signaling−Gq alpha and Go alpha mediated pathway
Insulin/growth factor signaling
PDGF signaling pathway
EGF receptor signaling pathway
Ras Pathway
Cellular signaling
Wnt signaling pathway
Immune function
B cell activation
Inflammation mediated by chemokine and cytokine signaling pathway
T cell activation
Toll receptor signaling pathway
0 1 2 3 4
−LOG10(p−value)
Genes
150
100
50
D
Shared FOXO targets
Biological process
Metabolism
metabolic process
primary metabolic process
nucleobase−containing compound metabolic process
RNA metabolic process
protein metabolic process
regulation of nucleobase−containing compound metabolic process
Transcription
transcription DNA−dependent
transcription from RNA polymerase II promoter
mRNA processing
chromatin organization
mRNA splicing, via spliceosome
General cellular processes
organelle organization
0 10 20 30 40 50
−LOG10(p−value)
Genes
1500
1000
500
E
Core FOXO targets
Biological process
Metabolism
nucleobase−containing compound metabolic process
metabolic process
primary metabolic process
RNA metabolic process
regulation of nucleobase−containing compound metabolic process
General cellular processes
organelle organization
regulation of biological process
Transcription
chromatin organization
regulation of transcription from RNA polymerase II promoter
transcription from RNA polymerase II promoter
transcription, DNA−dependent
Cell cycle
mitosis
0.0 2.5 5.0 7.5 10.0 12.5
−LOG10(p−value)
Genes
250
150
50

Figure S3

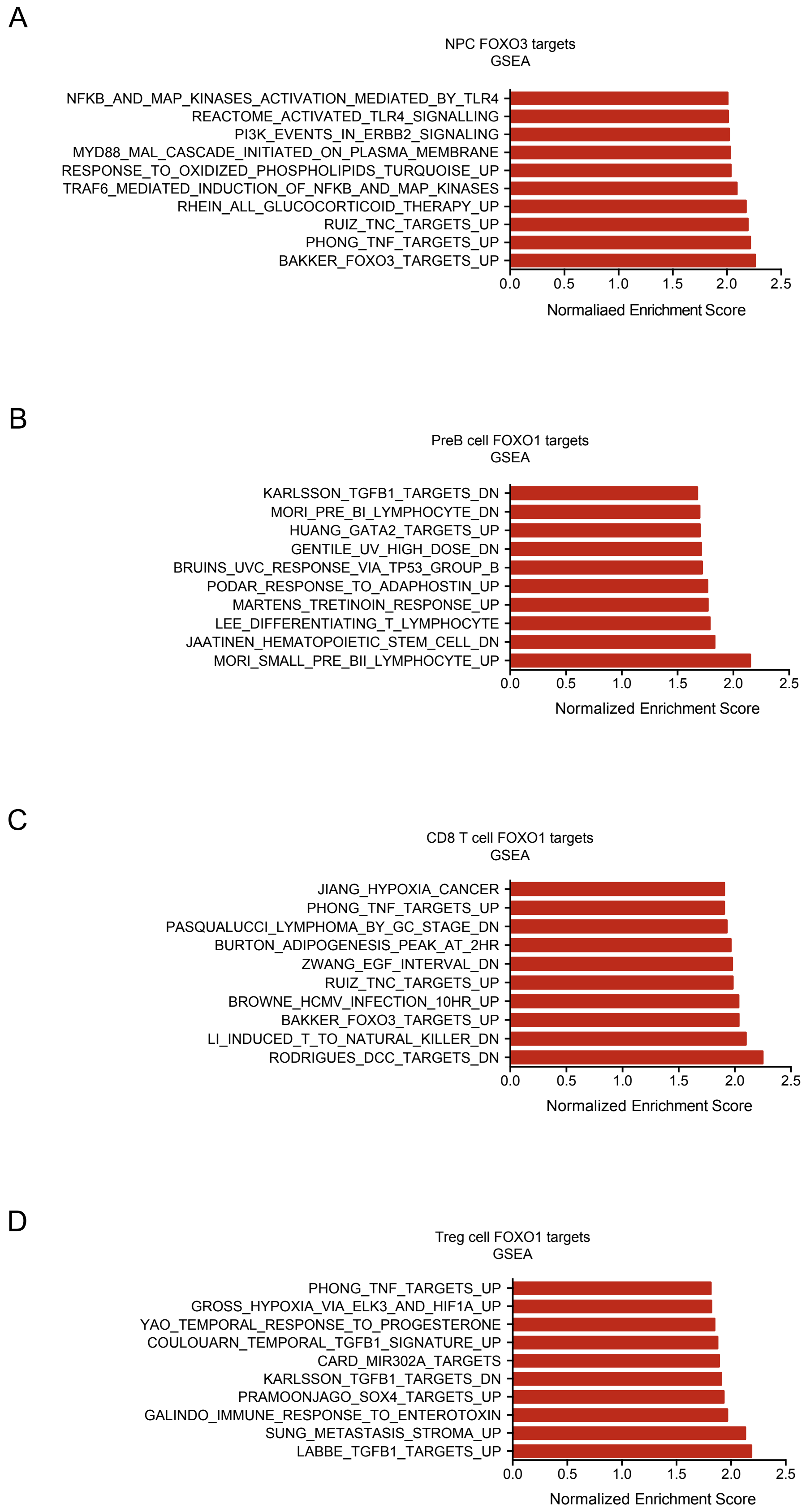
A
NPC FOXO3 targets
GSEA
NFKB_AND_MAP_KINASES_ACTIVATION_MEDIATED_BY_TLR4
REACTOME_ACTIVATED_TLR4_SIGNALLING
PI3K_EVENTS_IN_ERBB2_SIGNALING
MYD88_MAL_CASCADE_INITIATED_ON_PLASMA_MEMBRANE
RESPONSE_TO_OXIDIZED_PHOSPHOLIPIDS_TURQUOISE_UP
TRAF6_MEDIATED_INDUCTION_OF_NFKB_AND_MAP_KINASES
RHEIN_ALL_GLUCOCORTICOID_THERAPY_UP
RUIZ_TNC_TARGETS_UP
PHONG_TNF_TARGETS_UP
BAKKER_FOXO3_TARGETS_UP
0.0
0.5
1.0
1.5
2.0
2.5
Normaliaed Enrichment Score
B
PreB cell FOXO1 targets
GSEA
KARLSSON_TGFB1_TARGETS_DN
MORI_PRE_BI_LYMPHOCYTE_DN
HUANG_GATA2_TARGETS_UP
GENTILE_UV_HIGH_DOSE_DN
BRUINS_UVC_RESPONSE_VIA_TP53_GROUP_B
PODAR_RESPONSE_TO_ADAPHOSTIN_UP
MARTENS_TRETINOIN_RESPONSE_UP
LEE_DIFFERENTIATING_T_LYMPHOCYTE
JAATINEN_HEMATOPOIETIC_STEM_CELL_DN
MORI_SMALL_PRE_BII_LYMPHOCYTE_UP
0.0
0.5
1.0
1.5
2.0
2.5
Normalized Enrichment Score
C
CD8 T cell FOXO1 targets
GSEA
JIANG_HYPOXIA_CANCER
PHONG_TNF_TARGETS_UP
PASQUALUCCI_LYMPHOMA_BY_GC_STAGE_DN
BURTON_ADIPOGENESIS_PEAK_AT_2HR
ZWANG_EGF_INTERVAL_DN
RUIZ_TNC_TARGETS_UP
BROWNE_HCMV_INFECTION_10HR_UP
BAKKER_FOXO3_TARGETS_UP
LI_INDUCED_T_TO_NATURAL_KILLER_DN
RODRIGUES_DCC_TARGETS_DN
0.0
0.5
1.0
1.5
2.0
2.5
Normalized Enrichment Score
D
Treg cell FOXO1 targets
GSEA
PHONG_TNF_TARGETS_UP
GROSS_HYPOXIA_VIA_ELK3_AND_HIF1A_UP
YAO_TEMPORAL_RESPONSE_TO_PROGESTERONE
COULOUARN_TEMPORAL_TGFB1_SIGNATURE_UP
CARD_MIR302A_TARGETS
KARLSSON_TGFB1_TARGETS_DN
PRAMOONJAGO_SOX4_TARGETS_UP
GALINDO_IMMUNE_RESPONSE_TO_ENTEROTOXIN
SUNG_METASTASIS_STROMA_UP
LABBE_TGFB1_TARGETS_UP
0.0
0.5
1.0
1.5
2.0
2.5
Normalized Enrichment Score

Figure S4

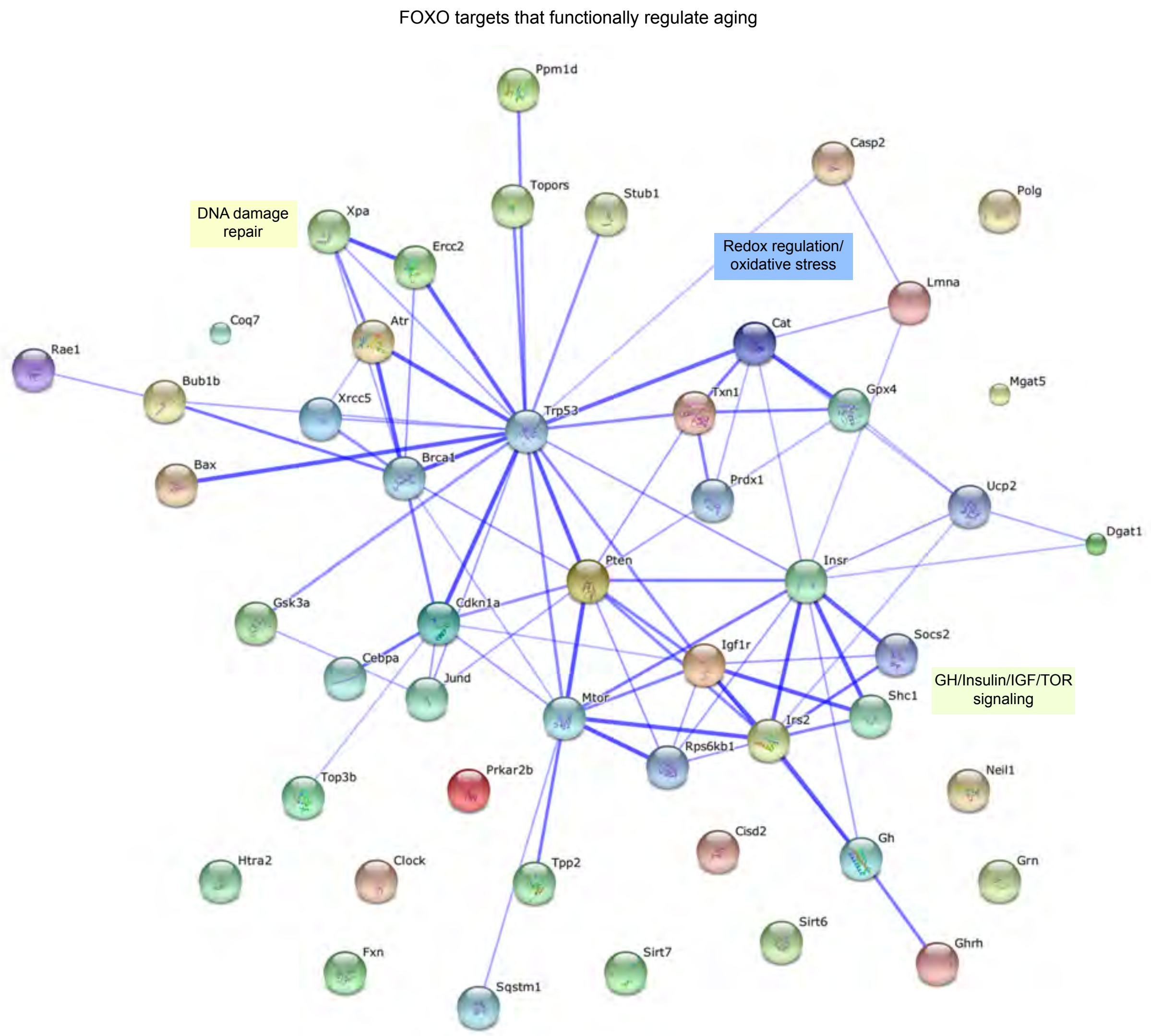

FOXO targets that functionally regulate aging
DNA damage repair
Redox regulation/ oxidative stress
GH/Insulin/IGF/TOR signaling
Ppm1d
Topors
Stub1
Casp2
Polg
Xpa
Ercc2
Lmna
Coq7
Atr
Cat
Rae1
Mgat5
Bub1b
Xrcc5
Txn1
Gpx4
Trp53
Bax
Brca1
Prdx1
Ucp2
Dgat1
Pten
Insr
Gsk3a
Cdkn1a
Socs2
Igf1r
Cebpa
Jund
Shc1
Mtor
Irs2
Rps6kb1
Neil1
Top3b
Prkar2b
Cisd2
Gh
Htra2
Clock
Tpp2
Grn
Sirt6
Fxn
Sirt7
Ghrh
Sqstm1

Figure S5

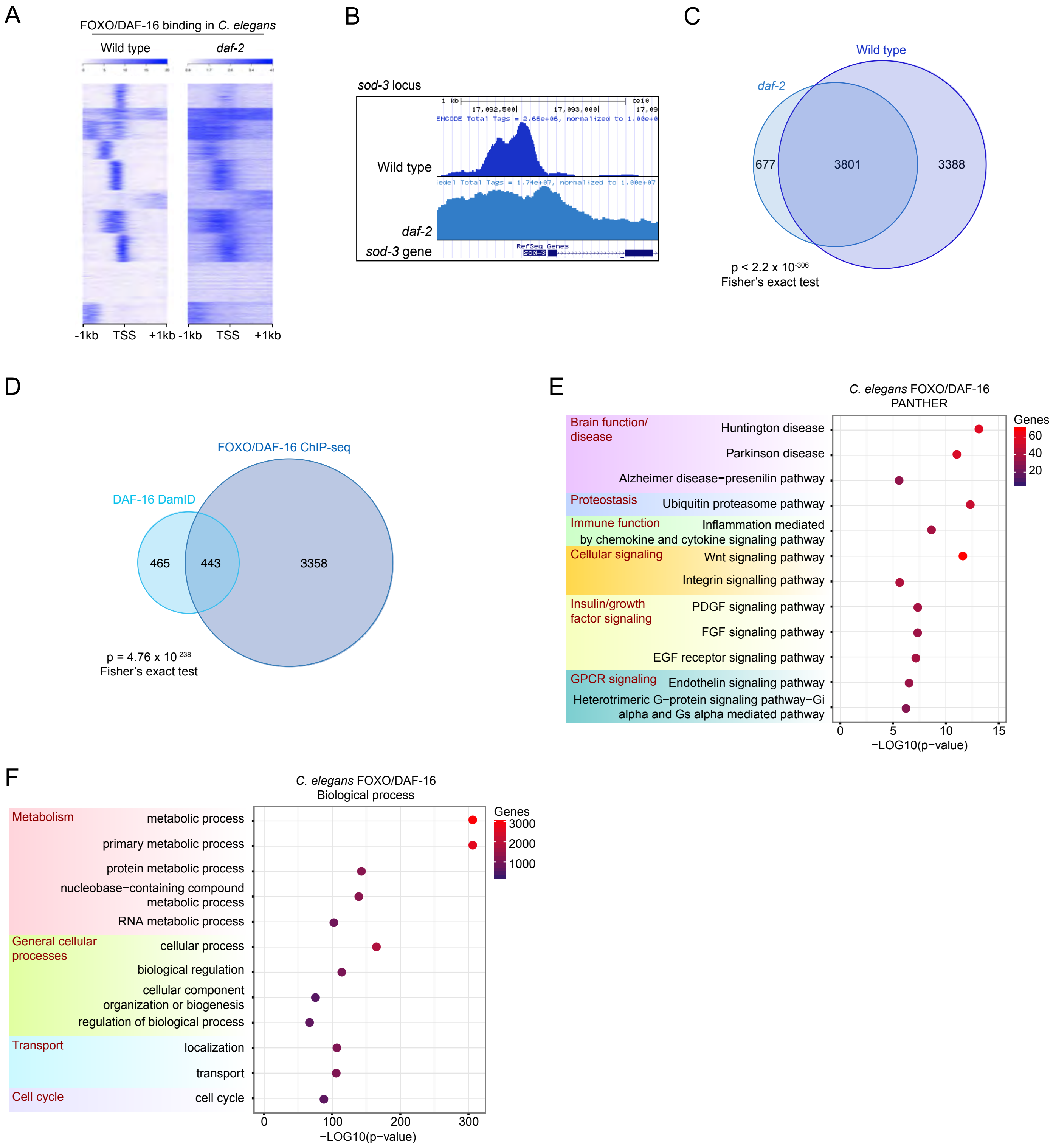

A
FOXO/DAF-16 binding in C. elegans
Wild type
daf-2
-1kb
TSS
+1kb
-1kb
TSS
+1kb
B
sod-3 locus
Wild type
daf-2
sod-3 gene
C
Wild type
daf-2
677
3801
3388
p < 2.2 x 10-306
Fisher's exact test
D
FOXO/DAF-16 ChIP-seq
DAF-16 DamID
465
443
3358
p = 4.76 x 10-238
Fisher's exact test
E
C. elegans FOXO/DAF-16
PANTHER
Brain function/ disease
Huntington disease
Parkinson disease
Alzheimer disease−presenilin pathway
Proteostasis
Ubiquitin proteasome pathway
Immune function
Inflammation mediated by chemokine and cytokine signaling pathway
Cellular signaling
Wnt signaling pathway
Integrin signalling pathway
Insulin/growth factor signaling
PDGF signaling pathway
FGF signaling pathway
EGF receptor signaling pathway
GPCR signaling
Endothelin signaling pathway
Heterotrimeric G−protein signaling pathway−Gi alpha and Gs alpha mediated pathway
Genes
60
40
20
0
5
10
15
−LOG10(p−value)
F
C. elegans FOXO/DAF-16
Biological process
Metabolism
metabolic process
primary metabolic process
protein metabolic process
nucleobase−containing compound metabolic process
RNA metabolic process
General cellular processes
cellular process
biological regulation
cellular component organization or biogenesis
regulation of biological process
Transport
localization
transport
Cell cycle
cell cycle
Genes
3000
2000
1000
0
100
200
300
−LOG10(p−value)

Figure S6

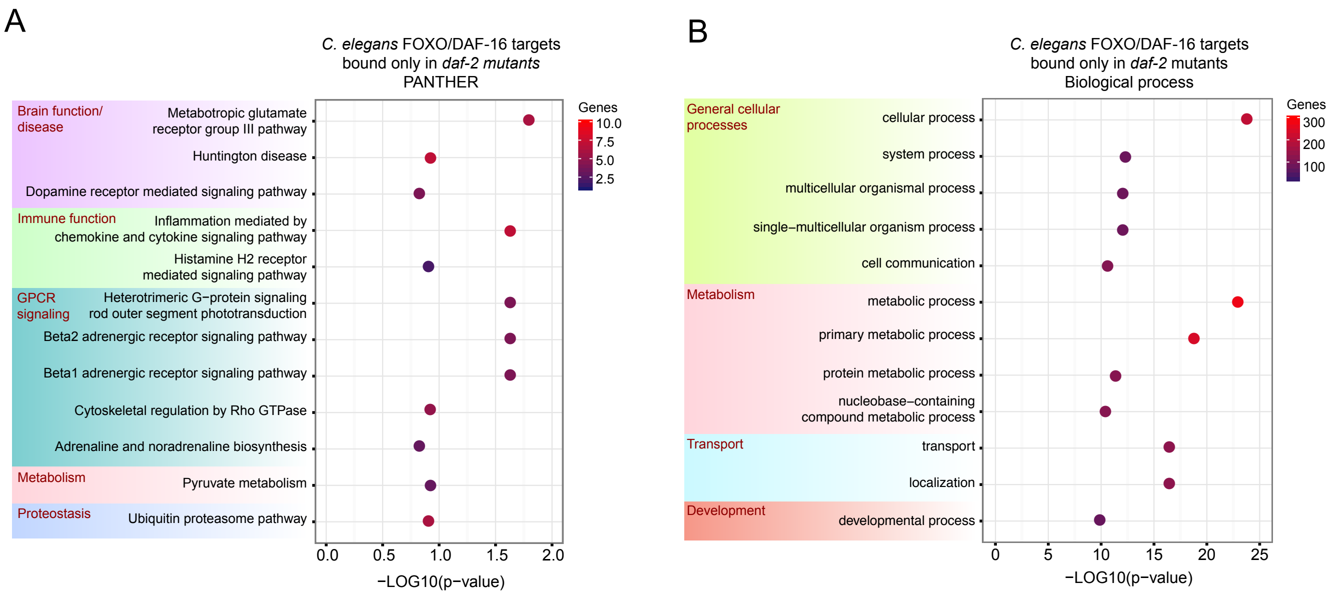

A
C. elegans FOXO/DAF-16 targets bound only in daf-2 mutants PANTHER
Brain function/ disease
Metabotropic glutamate receptor group III pathway
Huntington disease
Dopamine receptor mediated signaling pathway
Immune function
Inflammation mediated by chemokine and cytokine signaling pathway
Histamine H2 receptor mediated signaling pathway
GPCR signaling
Heterotrimeric G−protein signaling rod outer segment phototransduction
Beta2 adrenergic receptor signaling pathway
Beta1 adrenergic receptor signaling pathway
Cytoskeletal regulation by Rho GTPase
Adrenaline and noradrenaline biosynthesis
Metabolism
Pyruvate metabolism
Proteostasis
Ubiquitin proteasome pathway
0.0 0.5 1.0 1.5 2.0
−LOG10(p−value)
Genes
10.0
7.5
5.0
2.5
B
C. elegans FOXO/DAF-16 targets bound only in daf-2 mutants Biological process
General cellular processes
cellular process
system process
multicellular organismal process
single−multicellular organism process
cell communication
Metabolism
metabolic process
primary metabolic process
protein metabolic process
nucleobase−containing compound metabolic process
Transport
transport
localization
Development
developmental process
0 5 10 15 20 25
−LOG10(p−value)
Genes
300
200
100

Figure S7

FOXO targets that functionally regulate aging in mouse

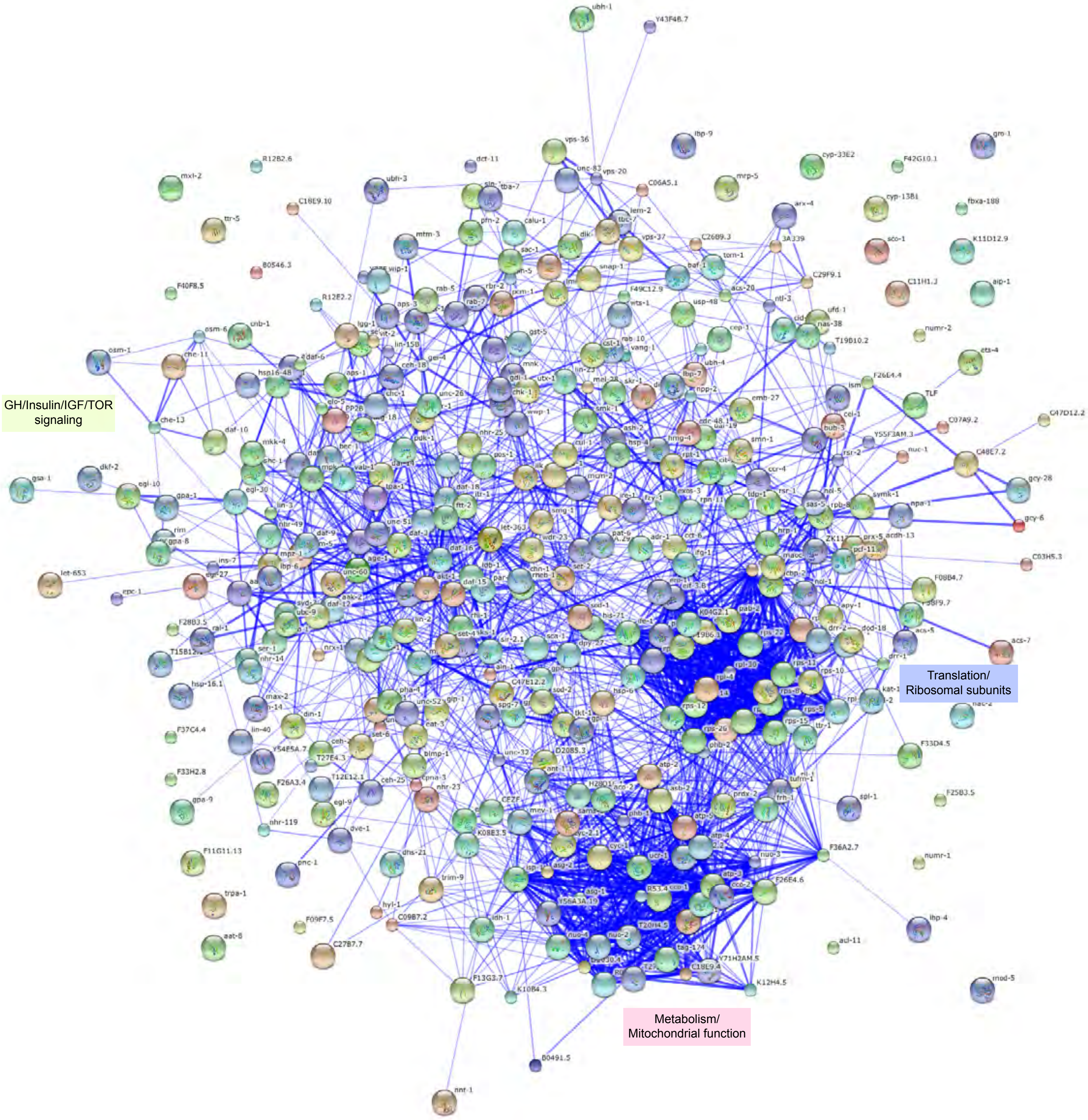

Figure S8

Conserved (mouse-worm) FOXO binding at functional aging regulators in *C. elegans*

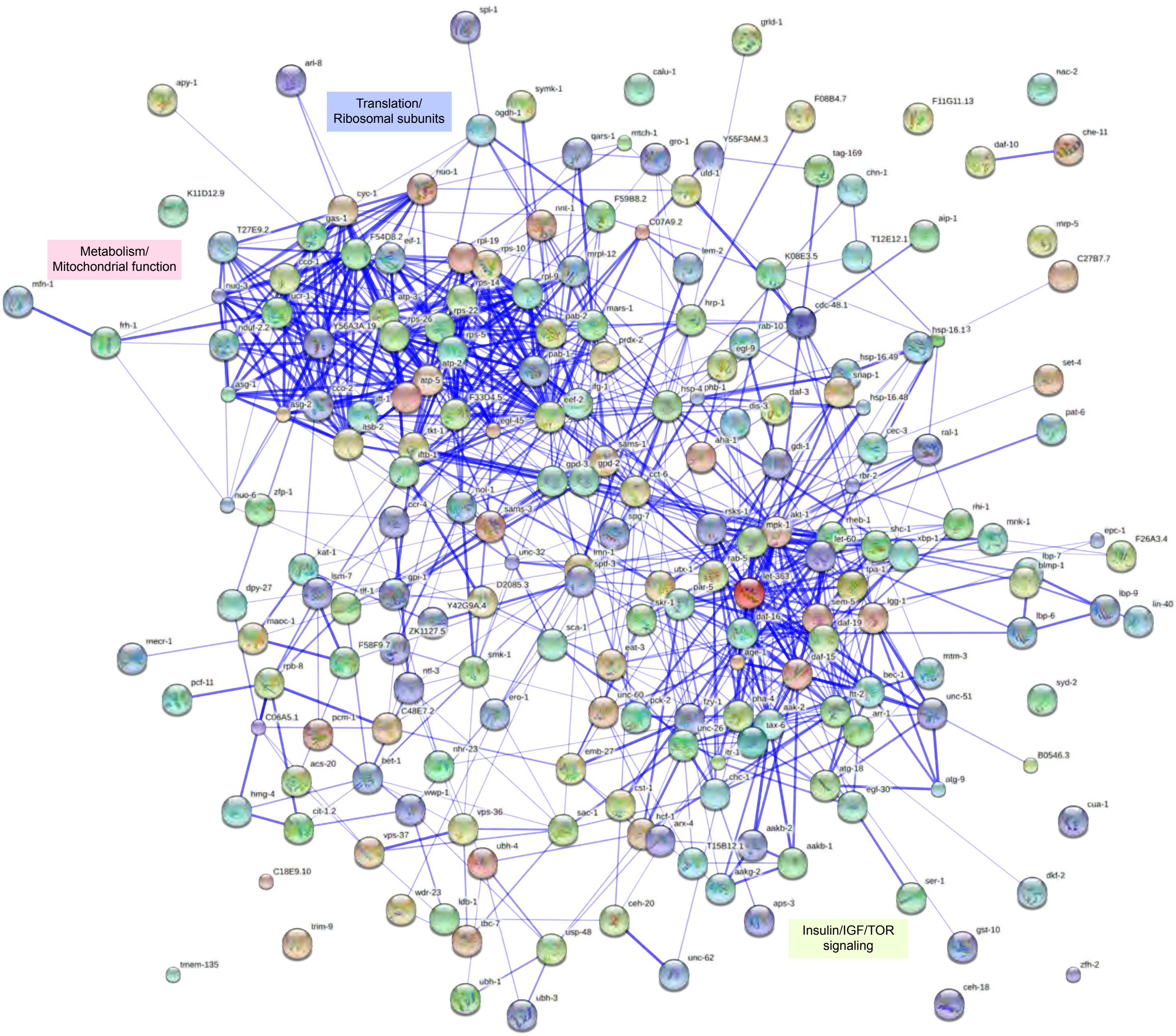

Figure S9

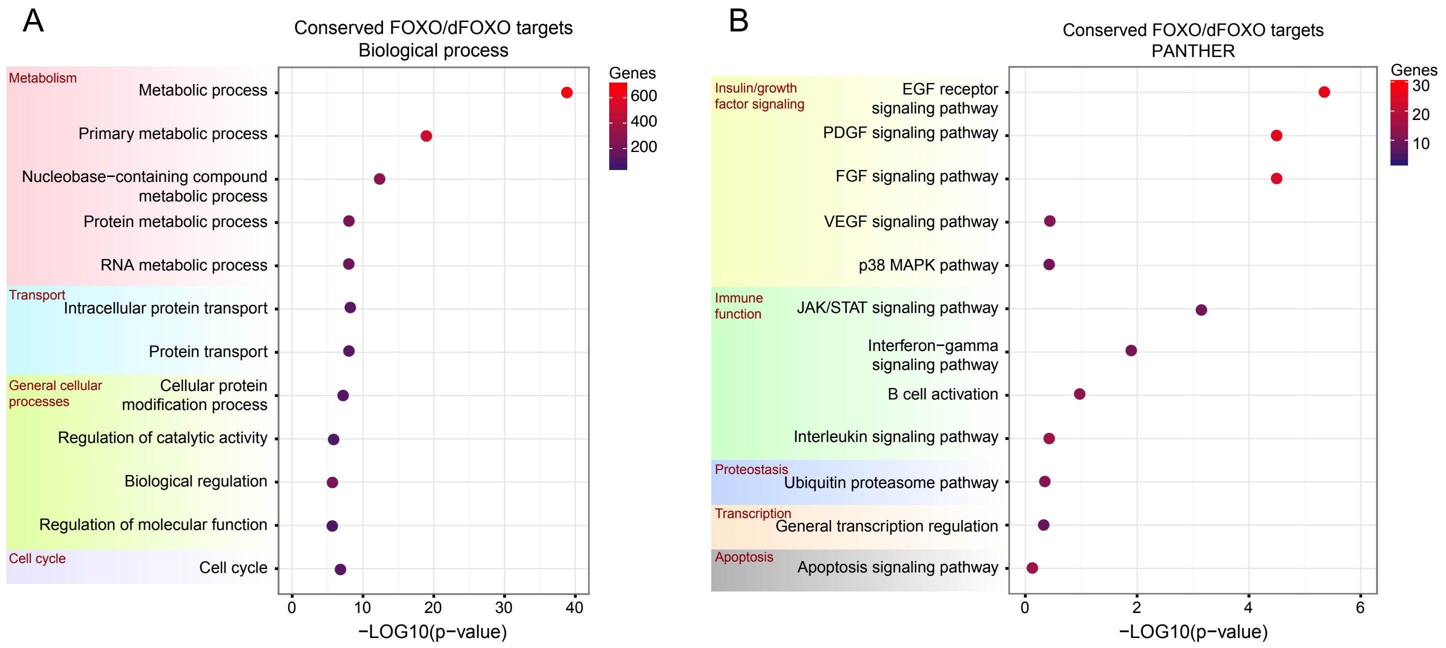
A
Conserved FOXO/dFOXO targets
Biological process
Metabolism
Metabolic process
Primary metabolic process
Nucleobase−containing compound metabolic process
Protein metabolic process
RNA metabolic process
Transport
Intracellular protein transport
Protein transport
General cellular processes
Cellular protein modification process
Regulation of catalytic activity
Biological regulation
Regulation of molecular function
Cell cycle
Cell cycle
0
10
20
30
40
−LOG10(p−value)
Genes
600
400
200
B
Conserved FOXO/dFOXO targets
PANTHER
Insulin/growth factor signaling
EGF receptor signaling pathway
PDGF signaling pathway
FGF signaling pathway
VEGF signaling pathway
p38 MAPK pathway
Immune function
JAK/STAT signaling pathway
Interferon−gamma signaling pathway
B cell activation
Interleukin signaling pathway
Proteostasis
Ubiquitin proteasome pathway
Transcription
General transcription regulation
Apoptosis
Apoptosis signaling pathway
0
2
4
6
−LOG10(p−value)
Genes
30
20
10

Figure S10

Network of FOXO/DAF-16
target genes that change in expression with age in *C. elegans*

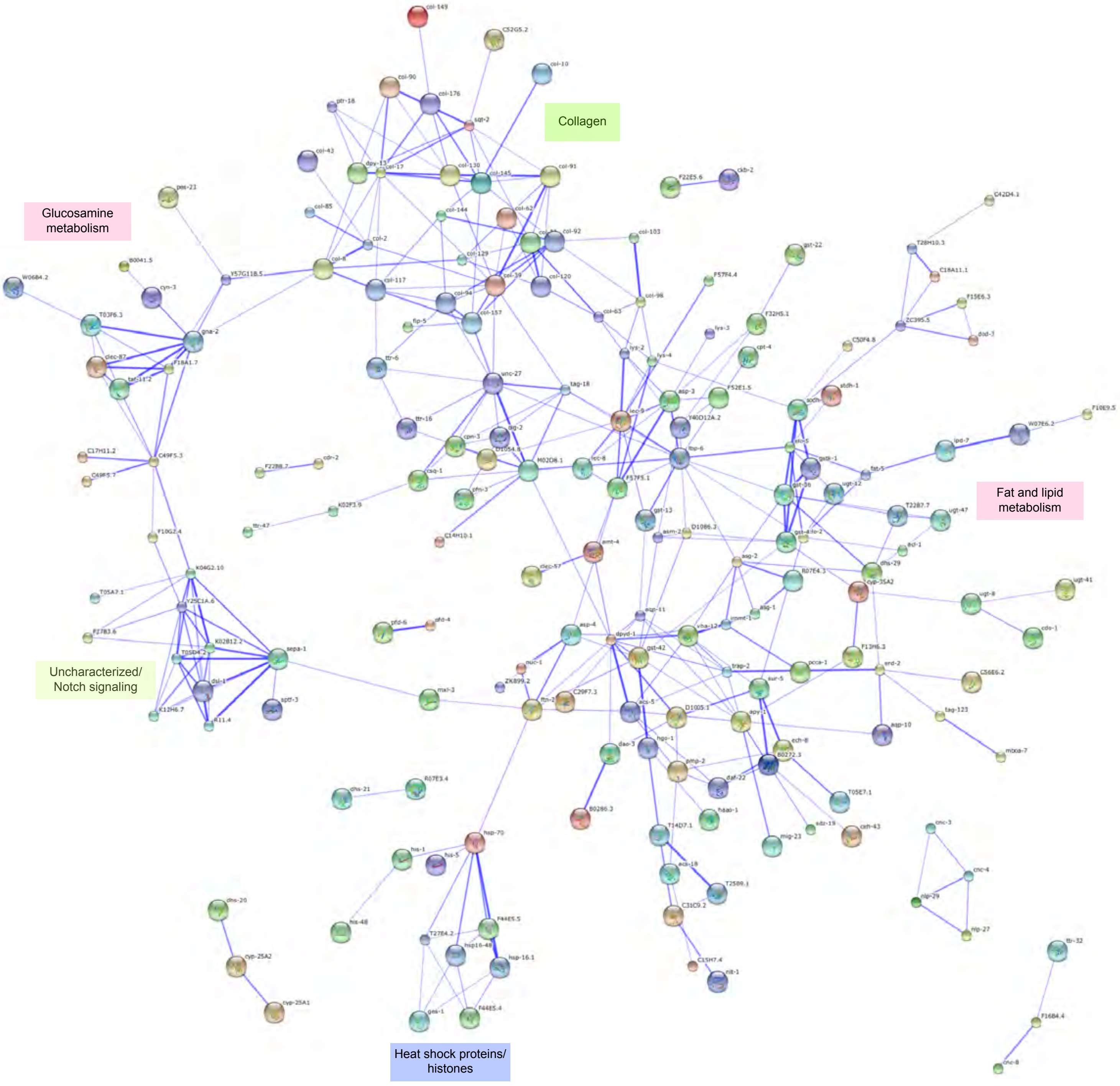

Figure S11

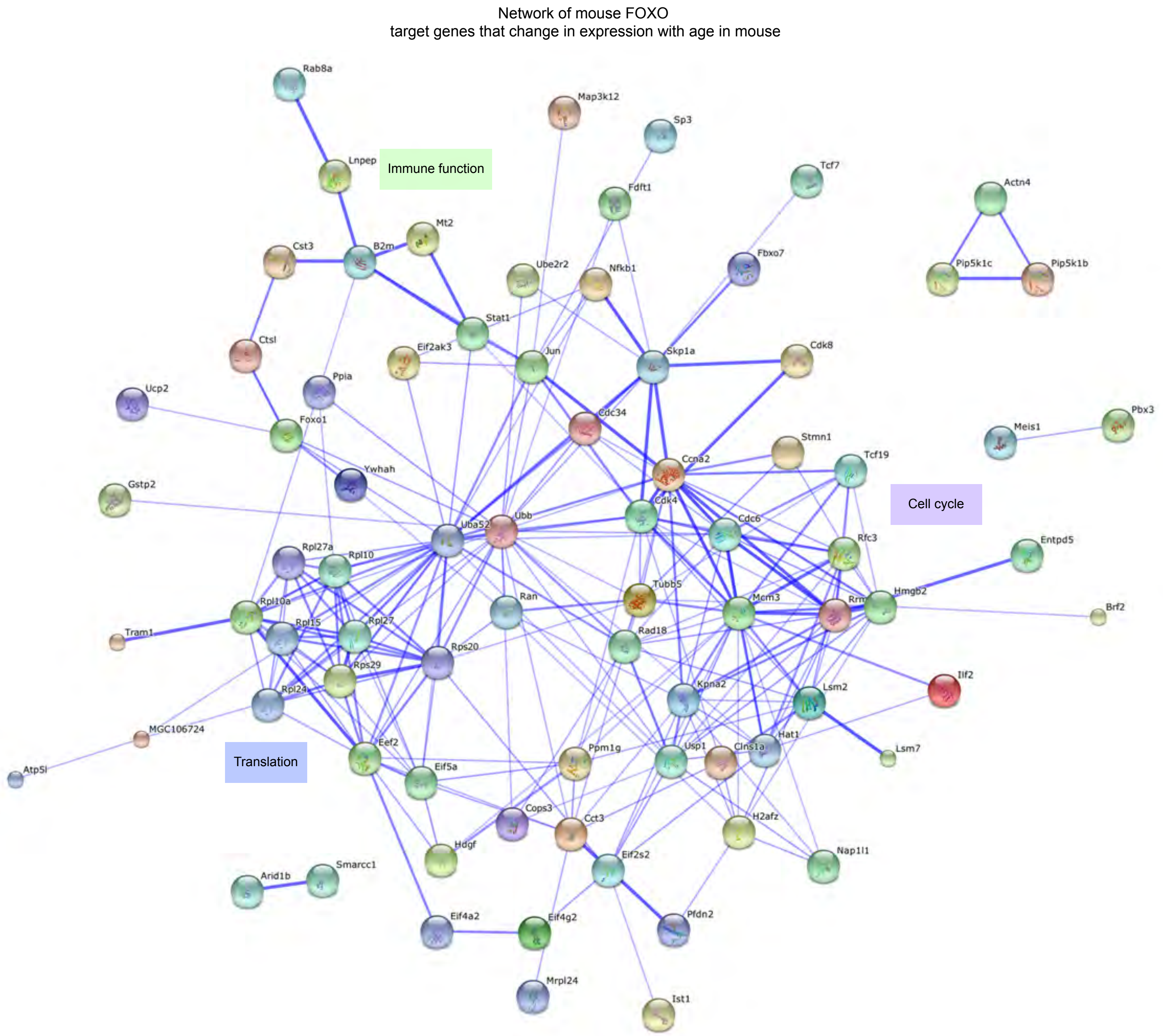
Network of mouse FOXO
target genes that change in expression with age in mouse
Immune function
Cell cycle
Translation
Rab8a
Lnpep
Map3k12
Sp3
Tcf7
Fdft1
Actn4
Pip5k1c
Pip5k1b
Cst3
B2m
Mt2
Ube2r2
Nfkb1
Fbxo7
Stat1
Ctsl
Eif2ak3
Jun
Skp1a
Cdk8
Ucp2
Ppia
Foxo1
Cdc34
Stmn1
Tcf19
Meis1
Pbx3
Ywhah
Ccna2
Gstp2
Cdk4
Uba52
Ubb
Cdc6
Rfc3
Entpd5
Rpl27a
Rpl10
Tubb5
Rpl10a
Ran
Mcm3
Hmgb2
Brf2
Tram1
Rpl15
Rpl27
Rad18
Rps20
Rps29
Ilf2
Rpl24
Kpna2
Lsm2
MGC106724
Hat1
Lsm7
Atp5i
Eef2
Usp1
Clns1a
Ppm1g
Eif5a
Cops3
H2afz
Cct3
Nap1l1
Hdgf
Eif2s2
Arid1b
Smarcc1
Eif4a2
Eif4g2
Pfdn2
Mrpl24
Ist1
