# Supplementary material for: Characterization of the direct targets of FOXO transcription factors throughout evolution
Source: Aging Cell. 2016 Apr 8;15(4):673–85. doi: 10.1111/acel.12479 (PMC4933671; doi:10.1111/acel.12479)
Supplement: Supplementary file 9 [file ACEL-15-673-s009.docx]

Suppl. Tables:

<http://tinyurl.com/webb-tables>
